# Distinct-Cluster Tree-Child Phylogenetic Networks and Possible Uses to Study Polyploidy

**DOI:** 10.1007/s11538-022-01084-6

**Published:** 2022-09-19

**Authors:** Stephen J. Willson

**Affiliations:** Department of Mathematics, Iowa State University, Ames, IA 50011 USA

**Keywords:** Phylogenetic network, Tree-child network, Normal network, CSD map, Polyploidy

## Abstract

As phylogenetic networks become more widely studied and the networks grow larger, it may be useful to “simplify” such networks into especially tractable networks. Recent results have found methods to simplify networks into normal networks. By definition, normal networks contain no redundant arcs. Nevertheless, there may be redundant arcs in networks where speciation events involving allopolyploidy occur. It is therefore desirable to find a different tractable class of networks that may contain redundant arcs. This paper proposes distinct-cluster tree-child networks as such a class, here abbreviated as DCTC networks. They are shown to have a number of useful properties, such as quadratic growth of the number of vertices with the number of leaves. A DCTC network is shown to be essentially a normal network to which some redundant arcs may have been added without losing the tree-child property. Every phylogenetic network can be simplified into a DCTC network depending only on the structure of the original network. There is always a CSD map from the original network to the resulting DCTC network. As a result, the simplified network can readily be interpreted via a “wired lift” in which the original network is redrawn with each arc represented in one of two ways.

## Introduction

A (rooted) phylogenetic tree is a tree in which the vertices correspond to biological species, the leaves are extant species, and the branchings correspond to speciation events, usually by mutation. Recently there has been increased interest in speciation events such as hybridization and lateral gene transfer which are not modeled well using trees (Delwiche and Palmer 1996; Doolittle and Bapteste 2007; Inagaki et al. 2002). Hence, there is interest in phylogenetic networks in which some nodes may have more than one parent. Overviews of phylogenetic networks may be found in Moret et al. (2004), Huson et al. (2010), and Steel (2016).

There are various interesting classes of networks that have been investigated. Tree-child networks (Cardona et al. 2009) are those such that each vertex not a leaf has a child with in-degree one, called a *tree-child*. Normal networks (Willson 2010) are also of interest; they are tree-child with the additional property that they have no redundant arcs. A *redundant* arc, sometimes called a short-cut, is an arc (*u*, *v*) such that there is another directed path from *u* to *v* that does not include (*u*, *v*). More details are given in Sect. 2.

A vertex *v* is *visible to a leaf x* provided every path from the root to *x* contains the vertex *v*. The vertex *v* is *visible* if it is visible to some leaf (Francis et al. 2021). If *v* is visible to a leaf *x*, then the genome at *v* can have a strong direct influence on the genomic inheritance at *x*. An example will be seen in Fig. 6.

As phylogenetic networks grow more complicated, their interpretation also becomes more complicated. Tree-child networks are particularly useful because every vertex is visible (see Cardona et al. 2009; Huson et al. 2010, and Sect. 4). Nevertheless, general tree-child networks are awkward since for a given number *n* of leaves, the number of vertices can be unbounded (Cardona et al. 2009).

Recently there has been interest in “simplifying” a general phylogenetic network into a normal network. Normal networks are tree-child but more tractable because the number of vertices grow at most quadratically with *n*. Suppose *N* is a phylogenetic network. Francis et al. (2021) have proposed a “normalization” which in this paper I will denote $\text {FHS}(N)$. A fast procedure *PhyloSketch* (Huson and Steel 2020) is available to compute $\text {FHS}(N)$. The current author (Willson 2022) has proposed a different construction of a normal network denoted $\text {Norm}(N)$.

One mechanism of speciation is polyploidization (Marcussen et al. 2015; Jones et al. 2013) in which the new species arises with twice the chromosomes, containing the whole genome of two parents. Such doubling of chromosomes is a very strong biological signal. Figure 4 of Marcussen et al. (2015) proposes 21 such allopolyploidization events, leading to 21 reticulations in the network. Of these, four have one ingoing arc a redundant arc. Perhaps that fact is not surprising; the two parental species would probably be very closely related. Both Francis et al. (2021) and Willson (2022) apply their methods to the network in Marcussen et al. (2015) to find related normal networks. These normal networks contain many fewer reticulations. By definition, a normal network contains no redundant arcs. If such speciation events involving redundant arcs are common, then insisting on normal networks is throwing out a lot of biological signal.

Moreover, Degnan (2018) argues that “ghost lineages” involving unsampled or extinct taxa can lead to reticulations involving redundant arcs or even parallel arcs. Similarly, the discussion in Francis et al. (2021) gives an example where an extinct lineage yields a redundant arc when only extant taxa are at the leaves. Finally, some of the scenarios in Fig. 1 of Jones et al. (2013) show redundant arcs.

It therefore might be useful to biologists to simplify phylogenetic networks into a different class of networks, still tractable, but that may contain redundant arcs. This paper proposes such a class, here called DCTC networks. They are formally introduced in Sect. 4.

Roughly, DCTC networks are defined by two properties:

1. No two vertices have the same cluster (except possibly a leaf and its parent) so they have “distinct clusters,” abbreviated DC.
2. The network is tree-child, abbreviated TC.

Since they have both properties, they are called *distinct-cluster tree-child networks*, or, more briefly, DCTC networks.

We shall see that a DCTC network *N* has additional interesting properties which in many ways resemble those of normal networks. In particular, a DCTC network satisfies the following:

(3) Suppose *N* has *n* leaves. Then the number of vertices of *N* is at most $(n^2 + n + 2)/2$. This upper bound is shown to be tight.
(4) Every vertex is visible.
(5) The number of hybrid vertices is at most $n-2$.
(6) For every vertex *v* with out-degree at least 2, there exist two distinct leaves *x*, *y* such that *v* is the most recent common ancestor of *x* and *y*.

Property (4) is true of all tree-child networks (Cardona et al. 2009) hence for all normal and DCTC networks; Properties (5) and (6) are also true of normal networks by results in Steel (2016) and Willson (2010), respectively. The estimate in Property (3) is very similar to a result for normal networks (Willson 2010), for which (if no vertex has out-degree one) the number of vertices is at most $(n^2+n)/2$. By contrast, if a TC network has *n* leaves, then the number of vertices can be unbounded. If a DC network has *n* leaves, then the number of vertices is bounded above by $2^n+n$. It is interesting that a DCTC network with both conditions can have at most $O(n^2)$ vertices.

The connection with normal networks is further studied in Sect. 6, where a DCTC network is shown essentially to be a normal network to which may have been added some redundant arcs while retaining the tree-child property. Moreover, in Sect. 8 we show that normal networks also can be easily modified into DCTC networks, although without redundant arcs.

In Willson (2012) and later (Willson 2022), the author studied CSD maps from one network to another. The topic will be reviewed in Sect. 3. Briefly, a CSD map $\psi :N\rightarrow N'$ consists of a surjective map $\psi :V(N)\rightarrow V(N')$ on the vertex sets with interesting properties concerning the arcs. They often correspond to some kind of “simplification” of *N*. Suppose $\psi :N\rightarrow N'$ is a CSD map. In that situation, the simplified network $N'$ can be visualized using a *wired lift of* $N'$ *into* *N* (initially defined in Willson (2012) and considerably extended in Willson (2022)). This wired lift redraws *N* including every arc, but each arc is drawn differently in one of a small number of ways so that $N'$ can be recognized in the modified drawing of *N*. Moreover, there is a path from $\psi (u)$ to $\psi (v)$ in $N'$ if and only if there is a *g-path* from *u* to *v* in the wired lift diagram (Willson 2022). (See Sect. 3 for more details.) Such a diagram can make it easier to interpret the simplification.

Here is a rough statement of the final result in this paper, in Sect. 9. Suppose *N* is a phylogenetic network for which the leaf set is identified with a set *X*. There is a procedure that systematically finds a DCTC network (here called $\text {DCTC}(N)$) for which there is a CSD map $\psi :N\rightarrow \text {DCTC}(N)$. The network $\text {DCTC}(N)$ depends only on the structure of *N*. By Property (3), if *N* has *n* leaves, then $\text {DCTC}(N)$ has at most $(n^2+n+2)/2$ vertices, hence has bounded complexity. There is a wired lift of $\text {DCTC}(N)$ into *N*. The construction resembles that in Willson (2022) for normal networks. The objective is to find a network related to *N* with strong internal confirmation of features such as reticulations.

In Sect. 11, the procedure is applied to several examples, two with real data. In particular, Example 2 applies it to the network *N* of Marcussen et al. (2015), which studies allopolyploidization in ${ Viola}$. The resulting network does in fact retain many more reticulations than were in the normalizations found in Francis et al. (2021) or Willson (2022), and it contains many redundant arcs.

A concluding discussion section treats biological interpretations of $\text {DCTC}(N)$.

## Basic Notions

The properties assumed in this paper are the same as in Willson (2022), which may serve as a reference for more detail.

Briefly, suppose *X* is a finite set (typically a set of extant species in the biological applications). An *X-network *$N=(V,A,\rho ,\phi )$ is a finite acyclic directed graph (*V*, *A*) where *V* is a finite set of vertices and *A* is a finite set of arcs. There are no directed cycles, there are no loops (*a*, *a*), and there is at most one arc (*a*, *b*) for $a\ne b$. The *in-degree* of a vertex *v* in *N*, denoted *indeg*(*v*) or *indeg*(*v*; *N*) , is the number of arcs (*u*, *v*), i.e., the number of parents of *v*. The *out-degree* of a vertex *v*, denoted *outdeg*(*v*) , is the number of arcs (*v*, *u*) , i.e., the number of children of *v*. Here $\rho$ is a vertex of in-degree 0, called the *root*; it is the only vertex with in-degree 0. A *leaf* is a vertex $x \in V$ with out-degree 0. The map $\phi :X\rightarrow V$ is a one-to-one map with image the set of leaves.

Occasionally we may have to deal with *X*-networks except that cycles are possible. If that occurs we will explicitly specify that the network is *not necessarily acyclic*.

Except where specified otherwise, we assume that each leaf $\phi (x)$ for $x\in X$ is a vertex with in-degree 1 and hence has a unique parent, which is denoted *p*(*x*; *N*) or *p*(*x*). The arc of form $(p(x),\phi (x))$ for some $x\in X$ will be called the *x*-*arc*. If *x* is not specified, any such arc will be called an *X*-*arc*.

Note that we make no assumption about the network being binary. A vertex may have in-degree greater than 2 or out-degree greater than 2 or both.

A *path* in *N* from *a* to *b* is a sequence $a=u_0, u_1, \ldots , u_k=b$ of vertices such that for $0\le i<k$, $(u_i,u_{i+1})\in A$. Paths in *N* are thus directed. If there is a path from *u* to *v*, then we write $u \le v$, and $\le$ is a partial order of *V*. For every vertex *v*, it is true that $\rho \le v$. We may write $u < v$ to mean $u\le v$ and $u\ne v$.

Suppose *x* and *y* are distinct vertices. A vertex *u* is a *common ancestor of x and y* if $u\le x$ and $u\le y$. A vertex *v* is a *most recent common ancestor of x and y*, denoted $\text {mrca}(x,y)$ or sometimes $\text {mrca}(x,y;N)$, if *v* is a common ancestor of *x* and *y* and, in addition, for every common ancestor *u* of *x* and *y*, $u\le v$. A most recent common ancestor $\text {mrca}(x,y)$ need not exist, and an example will be given in Fig. 6. If a most recent common ancestor of *x* and *y* exists, it is unique.

A vertex *v* is *hybrid* or *reticulate* if $indeg(v) \ge 2$. A child *u* of *v* is a *tree-child* if $indeg(u) = 1$, so (*v*, *u*) is the only arc coming into *u*. A vertex *v* is *trivial* if $indeg(v)=outdeg(v) = 1$.

An arc (*a*, *b*) is *redundant* or a *short-cut* if there exists a path $a = u_0, u_1, \ldots ,$ $u_n = b$, $n\ge 2$, that does not contain the arc (*a*, *b*).

For each $v \in V$, we write $cl(v;N) = \{x \in X: v \le \phi (x)\}$. If *N* is understood, we may write instead *cl*(*v*). We call it the *cluster* of *v*. Note that $cl(\rho ) = X$ and for each $v\in V$, *cl*(*v*) is nonempty. It is immediate that if $u \le v$ then $cl(v) \subseteq cl(u)$. Let $Cl(N) = \{cl(v): v \in V\}$ be the set of clusters of *N*.

There are several types of *X*-networks which will be of interest:

An *X*-network is *successively cluster-distinct* (SCD) (Willson 2012) if each arc (*a*, *b*) satisfies that either

(i) $$cl(b)\subsetneq cl(a)$$, or
(ii) $(a,b)=(p(x),\phi (x))$ for some $x\in X$ such that $cl(p(x))=cl(\phi (x))=\{x\}$.

Thus, successive vertices have different clusters except possibly for the arc $(p(x),\phi (x))$ entering a leaf. The definition is slightly modified from Willson (2012) because of our requirement that each leaf $\phi (x)$ must have in-degree one.

An *X*-network *N* is *tree-child* (Cardona et al. 2009) if every vertex that is not a leaf has a tree-child.

An *X*-network $N = (V,A,\rho ,\phi )$ (possibly with hybrid leaves) is *regular* (Baroni et al. 2004) if

1. the cluster map $cl: V \rightarrow P(X)$ is one-to-one, where *P*(*X*) is the power set of *X*;
2. *N* has no redundant arcs; and
3. $$u \le v$$ iff $$cl(v) \subseteq cl(u)$$.

Note that because of (3), any regular network is SCD.

An *X*-network *N* is *normal* (Willson 2010) if

1. *N* is tree-child; and
2. *N* contains no redundant arc.

Since this paper studies approximation of one network by another, we utilize a numerical distance between two arbitrary networks with the same leaf-set for comparisons, as in Willson (2022). Let *N* and $N'$ be *X*-networks. One interesting way to compare them is their *Robinson–Foulds distance* $d_{RF}(N,N')$. Here $d_{RF}(N,N')$ is defined as the number of members of *Cl*(*N*) and $Cl(N')$ which are present in one but not both. This definition is an extension of the notion for trees given in Robinson and Foulds (1981). For certain classes of *X*-networks, $d_{RF}$ is a metric. As an example, for fixed *X*, it is a metric on the collection of regular *X*-networks (Baroni et al. 2004).

## Prior Results

This section states results from Willson (2022) that will be needed, especially in Sect. 9.

Let $N = (V,A,\rho ,\phi )$ and $N' = (V',A', \rho ', \phi ')$ be *X*-networks. A *connected surjective digraph* (CSD) map (Willson 2012) $\psi : N \rightarrow N'$ is a map $\psi : V \rightarrow V'$ such that

1. $$\psi $$ is surjective.
2. For each arc $(u,v) \in A$, either $\psi (u) = \psi (v)$ or else $(\psi (u), \psi (v)) \in A'$. In the latter case, we may write $\psi (u,v)=(\psi (u),\psi (v))$. (Thus, $\psi$ is a *digraph map*).
3. For each $x \in X$, $\psi ( \phi (x)) = \phi '(x)$. More simply, $\psi (x) = x$.
4. $$\psi (\rho ) = \rho '$$.
5. For each $(u',v') \in A'$ there exists *u*, *v* in *V* such that $\psi (u) = u'$, $\psi (v)=v'$, and $(u,v) \in A$.
6. For each $v' \in V'$, $\psi ^{-1}(v')$ consists of the vertices of a connected subgraph of *N*. Thus, in the undirected graph *Und*(*N*) of *N*, if $W = \psi ^{-1}(v')$, the induced subgraph with vertex set *W* and edge set $\{\{u,v\}: \psi (u)=\psi (v)=v', (u,v) \in A$ or $(v,u) \in A\}$ is connected.

Note that if $u\le v$ in *N* and $\psi :N \rightarrow N'$ is a CSD map, then $\psi (u) \le \psi (v)$ in $N'$.

Let $N = (V,A,\rho ,\phi )$ and $N' = (V',A', \rho ', \phi ')$ be *X*-networks. A CSD-map $\psi : N \rightarrow N'$ is *leaf-preserving* if for each $x\in X$

(7) $u=\phi (x)$ is the only vertex in *V* such that $\psi (u)=\phi '(x)$, so $\psi ^{-1}(\phi '(x))=\{\phi (x)\}$; and
(8) the *x*-arc $(p(x),\phi (x))\in A$ is taken to the *x*-arc $(\psi (p(x)),\phi '(x))$; thus $\psi (p(x;N))=p(x;N')$.

If $\psi _1: N \rightarrow N'$ and $\psi _2: N' \rightarrow N''$ are CSD maps, then it is proved in Willson (2012) that the composition $\psi = \psi _2 \circ \psi _1: N \rightarrow N''$ is also a CSD map. If both maps are leaf-preserving, then so is the composition.

Let $\psi :N\rightarrow N'$ be a leaf-preserving CSD map, and let $2^V$ denote the set of subsets of *V*. A *wired lift* of *f* (or of $N'$ into *N*) is a pair $(\psi ^{-1},E_1)$ where $\psi ^{-1}$ is the map $\psi ^{-1}:V'\rightarrow 2^V$ given by $\psi ^{-1}(v')$ and where $E_1\subseteq A$ satisfies the following two conditions:

1. For each arc $(u,v)\in E_1$, $\psi (u)\ne \psi (v)$ and $(\psi (u),\psi (v))\in A'$. Denote $\psi (u,v)=(\psi (u),\psi (v))$.
2. For every arc $(u',v')\in A'$, there exists $(u,v)\in E_1$ such that $\psi (u,v)=(u',v')$. We will say the arc (*u*, *v*) *represents* $(u',v')$ or is a *pre-arc* of $(u',v')$.

Call the members of $E_1$ the *representative arcs* since each represents an arc of $A'$.

Note that the collection of all $\psi ^{-1}(v')$ for $v'\in V'$ is a partition of *V*. Thus for all $v'\in V'$, $\psi ^{-1}(v')\ne \emptyset$; if $u'\ne v'$ are in $V'$, then $\psi ^{-1}(u')\cap \psi ^{-1}(v')=\emptyset$; and $\cup \psi ^{-1}(v') = V$ where the union is over all $v'\in V'$.

### Theorem 3.1

Willson (2022) Suppose *N* and $N'$ are *X*-networks and $\psi :N\rightarrow N'$ is a CSD map. If $E_1=\{(u,v)\in A: \psi (u)\ne \psi (v)\}$, then $(\psi ^{-1},E_1)$ is a wired lift.

**Fig. 1:**
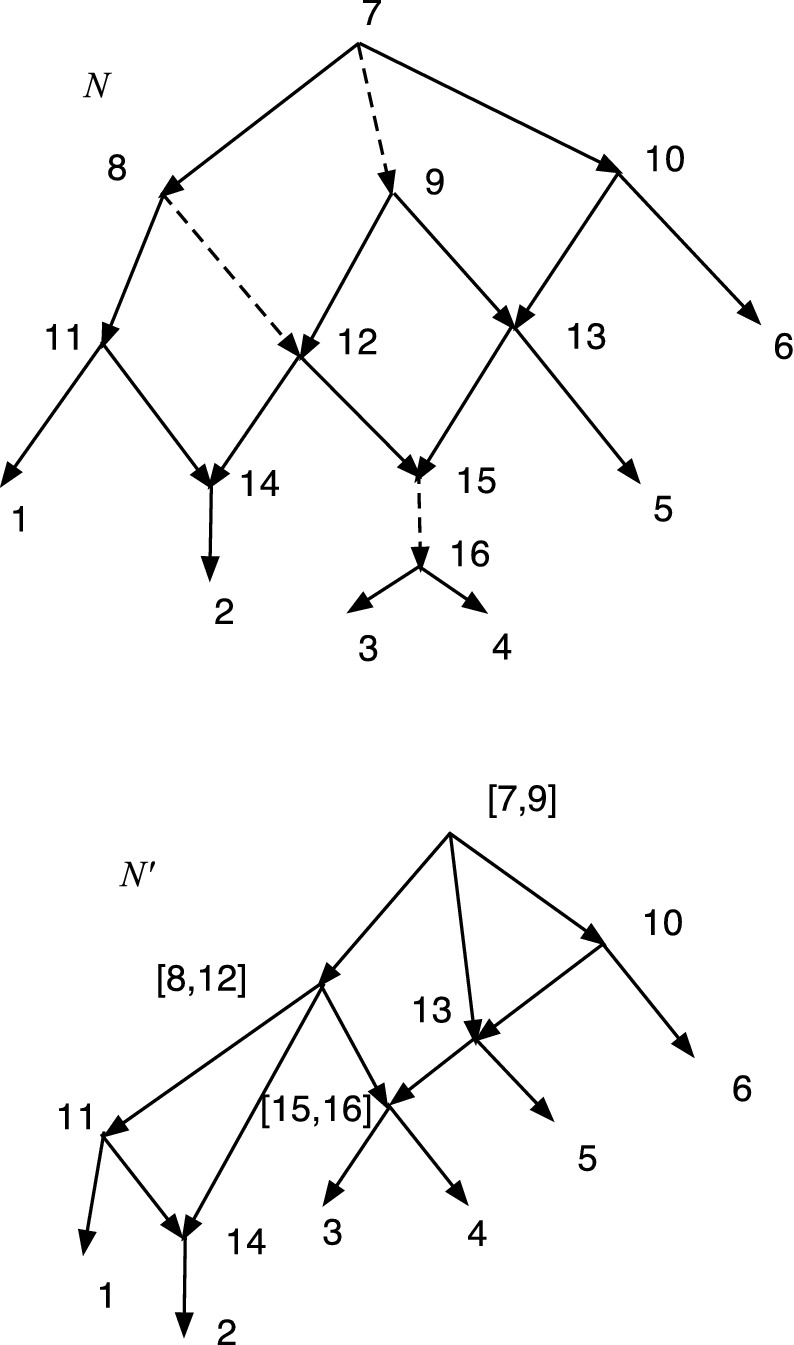
An example of a wired lift for a CSD map $\psi :N\rightarrow N'$. The upper graph shows the wired lift as a diagram of *N*. Dashed arcs indicate identification of the vertices and can be followed in either direction. Solid arcs must be followed in their direction. If all arcs were solid, then the upper diagram would be exactly *N*. The lower diagram shows $N'$. Note that, as indicated by the dashed arcs, 7 and 9 are identified into [7,9]. Similarly 8 and 12 are identified into [8,12] while 15 and 16 are identified into [15,16]. The map $\psi$ satisfies, for example, $\psi (8)=\psi (12)=[8,12]$, $\psi (7)=\psi (9)=[7,9]$, and $\psi (11)=11$

The wired lift $(\psi ^{-1},E_1)$ can be visualized using a diagram of *N*. An example is shown in Fig. 1. The diagram is exactly the diagram of *N* except that each arc may be solid or dashed. Suppose $N=(V,A,\rho ,\phi )$. For every arc $(u,v)\in A$ such that $\psi (u)\ne \psi (v)$ draw (*u*, *v*) a solid arrow if $(u,v)\in E_1$. For each arc $(u,v)\in A$ such that $\psi (u)=\psi (v)$ draw the arc as a dashed arrow. Dashed arcs make the sets $\psi ^{-1}(v')$ apparent in *N* and each vertex of $N'$ corresponds to a connected component of the dashed arcs. Each arc $(u',v')\in A'$ has at least one corresponding solid arc $(u,v)\in A$, justifying the word “lift.” The “wires” are the dashed arcs. In Willson (2022), the wired lifts for normal networks could contain three types of arcs–wide solid, thin solid, and dashed. For DCTC-networks, however, only two types are needed and for ease of visualization we choose solid and dashed. The solid arcs in this paper correspond to the wide solid arcs in Willson (2022), while the dashed arcs in this paper correspond to the thin solid arcs in the previous paper.

Let $N=(V,A,\rho ,\phi )$ and $N'=(V',A',\rho ',\phi ')$ be *X*-networks, with $\psi :V\rightarrow V'$ a CSD map, and suppose $(\psi ^{-1},E_1)$ is a wired lift of *f*. If *u* and *v* are in *V*, we say there is an *allowed step* from *u* to *v* if either $(u,v)\in E_1$, or ($(u,v)\in A$ and $f(u)=f(v)$), or ($(v,u)\in A$ and $f(u)=f(v)$). Note that the step either follows a solid arc in $E_1$ forwards or else follows a dashed arc, possibly forwards, possibly backwards.

A *generalized path* or *g*-path in *N* from *a* to *b* is a sequence $a=u_0,u_1, \ldots , u_k=b$ of vertices such that for $i=0,\ldots ,k-1$, there is an allowed step from $u_i$ to $u_{i+1}$.

In Fig. 1, let *N* denote the initial *X*-network and $N'$ be such that $\psi :N\rightarrow N'$ is a CSD map, where the upper part of Fig. 1 is the wired lift. If all arcs in the upper part of Fig. 1 were solid, then the figure would show *N*. Because of the dashed arcs, we see that $\psi (8)=\psi (12)$, $\psi (7)=\psi (9)$, and $\psi (15)=\psi (16)$. There is a g-path 12,8,11,1; hence, in $N'$ there is a path from $\psi (12)$ to $\psi (1)=1$. In *N*, there is clearly no path from 12 to 1. Note also that all children of 12 in *N* are hybrid. But $\psi (12)= [8,12]$ in $N'$ has the tree-child 11. In fact, $N'$ is tree-child.

### Theorem 3.2

Willson (2022) Let $N=(V,A,\rho ,\phi )$ and $N'=(V',A',\rho ',\phi ')$ be *X*-networks, with $\psi :N\rightarrow N'$ a CSD map. There is a path from $\psi (a)$ to $\psi (b)$ in $N'$ if and only if there is a *g*-path in *N* from *a* to *b*.

Let $N=(V,A,\rho ,\phi )$ be an *X*-network. A subset $K\subseteq A$ of arcs is *strongly closed* if it satisfies the following: Suppose there are vertices $a, u_0, u_1, \ldots , u_{2m}=b$ in *V* with $m \ge 1$ such that $a \sim _K u_0$, $(u_0,u_1)\in A$, $u_1\sim _K u_2$, $(u_2,u_3)\in A$, $u_3\sim _K u_4$, $\cdots$, $(u_{2m-2},u_{2m-1})\in A$, $u_{2m-1}\sim _K u_{2m}=b$, and in addition $a\sim _K b$. Then for *k* such that $0\le k\le m-1$ each of the arcs $(u_{2k},u_{2k+1})$ lies in *K*.

### Theorem 3.3

Willson (2022) Let $N=(V,A,\rho ,\phi )$ be an *X*-network and $D\subseteq A$ be a subset of arcs. There exists a unique $K\subseteq V$ such that

(i) $$D\subseteq K$$,
(ii) *K* is strongly closed, and
(iii) for every strongly closed $C\subseteq A$ such that $D\subseteq C$, it follows that $K\subseteq C$.

Thus, *K* is the unique minimal strongly closed subset of *A* containing *D*.

The subset *K* of the theorem is denoted *K*(*D*) and called the *strong closure of D*.

The following theorem summarizes the fundamental construction $M_D(N)$ described in Willson (2022). While (1), (2), and (3) were explicit, (4) was only implicit in Willson (2022).

### Theorem 3.4

Willson (2022) Suppose $N=(V,A,\rho ,\phi )$ is an *X*-network and $D\subseteq A$ contains no *X*-arc. There is a uniquely determined *X*-network $M_D(N)$ such that

1. There is a projection map $\psi :N\rightarrow M_D(N)$ which is a leaf-preserving CSD map.
2. For each arc $(a,b)\in D$, $\psi (a)=\psi (b)$.
3. If *K*(*D*) is the strong closure of *D*, then $K(D) = \{(a,b)\in A: \psi (a)=\psi (b)\}$.
4. Suppose $N'$ is an *X*-network, $f:N\rightarrow N'$ is a leaf-preserving CSD map, and for each arc $(a,b)\in D$, $f(a)=f(b)$. If $(u,v)\in A$ and $\psi (u)=\psi (v)$, then it follows $f(u)=f(v)$.

The idea of $M_D(N)$ is relatively simple. The set *D* consists of a list of arcs in *N*. For each arc $(a,b)\in D$, we contract the arc in *N* to a point. If these contractions result in any directed cycles, we contract the arcs in any such cycle. The result is $M_D(N)$, which is shown in Willson (2022) to be a well-defined acyclic network. Since $M_D(N)$ is obtained by contracting certain arcs of *N*, it is a kind of quotient graph of *N*.

### Proof

Parts (1), (2), and (3) are directly from Willson (2022). We prove Part (4), using more details from Willson (2022).

Let $E=\{(u,v)\in A: f(u)=f(v)\}$. I claim that $K(D) \subseteq E$. By hypothesis, $D\subseteq E$. We now return to the proof of Theorem 3.3 (Theorem 3.7 of Willson (2022)). That proof constructs a sequence $D_0, D_1, \cdots$ of subsets of *A* that starts with $D_0=D$ and ends with *K*(*D*). An easy inductive argument shows that each $D_i$ satisfies $D_i\subseteq E$. Hence, $K(D)\subseteq E$.

Now we may complete the proof of Part (4). Suppose $(u,v)\in A$ and $\psi (u)=\psi (v)$. By Part (3), $(u,v)\in K(D)$. But $K(D)\subseteq E$. Hence, $f(u)=f(v)$. $\square$

Note that (4) shows that if $f:N\rightarrow N'$ is a CSD map which contracts the arcs of *D* and $N'$ is an acyclic *X*-network, then all the identifications in $M_D(N)$ occur also in $N'$. Thus, all the identifications in $M_D(N)$ are needed to obtain an acyclic *X*-network that contracts the members of *D*.

As an application, there is the following result:

### Theorem 3.5

Willson (2022) Let *N* be an acyclic *X*-network. There is an *X*-network $\text {SCD}(N)$ such that

1. $\text {SCD}(N)$ is successively-cluster-distinct (SCD).
2. There is a leaf-preserving CSD map $\psi :N\rightarrow \text {SCD}(N)$.
3. $\text {SCD}(N)$ contains no trivial vertices.
4. $$Cl(\text {SCD}(N))=Cl(N)$$, so $$d_{RF}(N,\text {SCD}(N))=0$$.

Essentially, the computation of $\text {SCD}(N)$ contracts to a single vertex each arc (*a*, *b*) such that $cl(a)=cl(b)$. Special attention is given to the case where *b* is a leaf to ensure that no leaf becomes hybrid.

## Basic Properties of *X*-Networks that are Both Distinct-Cluster and Tree-Child

An *X*-network *N* is *distinct-cluster* or *DC* if, whenever *u* and $v\in V$ satisfy $cl(u)=cl(v)$, then either $u=v$ or else one of *u* and *v* is a leaf $\phi (x)$ for some $x\in X$ and the other is *p*(*x*) where *p*(*x*) is hybrid with out-degree 1. Thus the only way to have $cl(u)=cl(v)$ is that either $u=v$ or $\{u,v\} = \{\phi (x), p(x)\}$ for some $x\in X$ such that *p*(*x*) is hybrid with out-degree 1. More briefly, the only way to have $cl(u)=cl(v)$ must involve both ends of the *x*-arc $(p(x),\phi (x))$ in the case where *p*(*x*) is hybrid with out-degree 1. It is immediate that if *p*(*x*) is hybrid with out-degree 1, then $cl(p(x))=cl(\phi (x))=\{x\}$. The definition is intended to modify slightly the idea that no two vertices have the same cluster so as to be consistent with our assumption that each leaf has in-degree one.

A path $u = u_0, u_1, \ldots , u_k = b$ is a *tree-child path* if, for $i=0,\ldots , k-1$, $(u_i, u_{i+1})\in A$ and $indeg(u_{i+1})=1$.

The following theorem restates some results in Cardona et al. (2009).

### Theorem 4.1

Suppose $N=(V,A,\rho ,\phi )$ is an *X*-network that is tree-child.

1. Given any vertex $v\in V$ that is not a leaf, there is a tree-child path from *v* to some leaf $\phi (x)$.
2. Assume there is a tree-child path $u = u_0, u_1, \ldots , u_k = b$ with $k\ge 1$. Then any path $v = v_0, v_1, \ldots , v_j=b$ satisfies that either $v=u_i$ for some $i>0$ or else $v\le u$.

### Theorem 4.2

Suppose $N=(V,A,\rho ,\phi )$ is an *X*-network that is both SCD and tree-child. Then *N* is distinct-cluster (DC).

### Proof

Suppose *u* and $v\in V$ satisfy $cl(u)=cl(v)$. If *u* is a leaf, say $u=\phi (x)$, then $cl(u)=\{x\}$. Then $v=p(x)$ by SCD and the claim is true. The same is true if *v* is a leaf. So we may assume that neither *u* nor *v* is a leaf. There is a tree-child path $P: u=v_0, v_1, \ldots , v_k=\phi (x)$ from *u* to a leaf $\phi (x)$ since *N* is tree-child, and hence $x\in cl(u)$. It follows that $x\in cl(v)$ so there is a path from *v* to $\phi (x)$. By Theorem 4.1, either *v* is a vertex of *P* or else $v\le u$. If $v=v_j$ for some $j>0$, then $cl(v_0)=cl(v_j)$ whence $cl(v_0)=cl(v_1)=\cdots =cl(v_j)$, contradicting that *N* is SCD. Thus $v \le u$. A symmetric argument proves $u\le v$. Hence $u=v$. $\square$

We will call an *X*-network *N* a *DCTC* *X*-network if it is acyclic, distinct-cluster (DC), and tree-child (TC).

### Corollary 4.3

An *X*-network that is SCD and tree-child is a DCTC *X*-network.

**Fig. 2:**
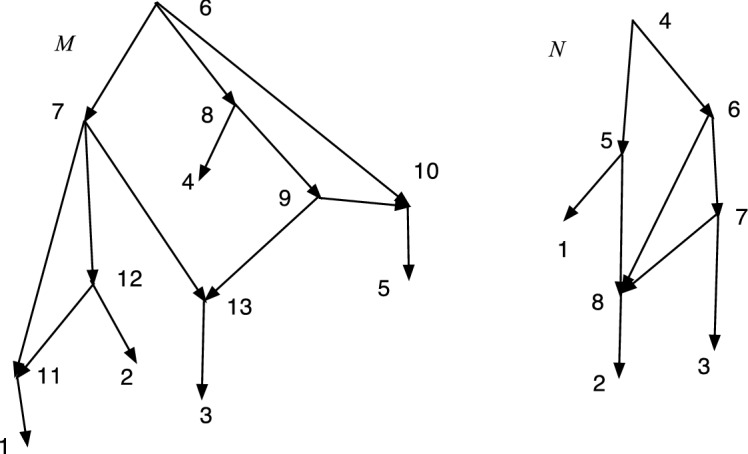
*M* is DC but not TC, while *N* is TC but not DC

In Fig. 2, *M* is DC but not TC since 9 has no tree-child. *N* is TC but not DC since $cl(6)=cl(7)=\{2,3\}$.

Any tree-child network with *n* leaves has at most $n-1$ hybrid vertices by results in Cardona et al. (2009). The following result is a slight improvement for DCTC networks. The bound $n-2$ is the same as for normal networks; see Steel (2016).

### Theorem 4.4

Suppose $N=(V,A,\rho ,\phi )$ is a DCTC *X*-network with *n* leaves. Then the number of hybrid vertices is at most $n-2$.

### Proof

Since *N* is TC, the root $\rho$ must have a tree child $c_1$. Since *N* is DC, $cl(\rho )\ne cl(c_1)$. Hence, $\rho$ must have a child $c_2$ such that $cl(c_2)$ is not contained in $cl(c_1)$. There is a path from $\rho$ to $c_2$. Choose a path from $\rho$ to $c_2$ of maximal length. The child *c* of $\rho$ on that path cannot be hybrid since the other parent cannot be $\rho$ and hence the path could be made longer. Hence, *c* is a tree-child. We cannot have $c=c_1$ because then $c\le c_2$ so $cl(c_2) \subseteq cl(c)= cl(c_1)$, a contradiction. Hence, *c* and $c_1$ are two distinct tree-children of $\rho$.

Choose a tree-path from $c_1$ to the leaf $\phi (x)$ and a tree-path from *c* to the leaf $\phi (y)$. I claim that $x\ne y$. Otherwise if $\phi (x)=\phi (y)$, by Theorem 3.1(2) either $c_1$ is on the path from *c* to $\phi (y)$ or $c_1 \le c$. The latter cannot happen since the path would have to go through $\rho$, the unique parent of *c*. Hence, $c_1$ lies on the path from *c* to $\phi (y)$. By a symmetric argument, *c* lies on the path from $c_1$ to $\phi (x)=\phi (y)$. Thus, *c* lies on a cycle, contradicting that *N* is acyclic.

Suppose the hybrid vertices are $h_1, h_2, \ldots , h_k$. From $h_i$, there is a tree-child path to some leaf $\phi (x_i)$. by a similar argument the members $x,y, x_1, \ldots , x_k$ are all distinct. Hence $k+2 \le n$, so $k\le n-2$. $\square$

Let $N=(V,A,\rho ,\phi )$ be an acyclic *X*-network. A vertex *v* is *visible* (Francis et al. (2021); Huson et al. (2010)) if there exists a leaf $\phi (x)$ for $x\in X$ such that every path from $\rho$ to $\phi (x)$ passes through *v*. For example, in *M* of Fig. 2 note that 9 is not visible since a path to 5 from the root 6 can pass through 10 and not 9; and a path to 3 from 6 can pass through 7 and not 9; moreover, 5 and 3 are the only leaf descendants of 9. On the other hand, 7 is visible since every path from 6 to 1 passes through 7.

As is pointed out in Francis et al. (2021) if a vertex *v* is not visible, then the evolutionary history of gene flow in the corresponding phylogenetic network may have bypassed *v* and the presence of *v* could have no genetic impact on any of the leaves. Hence, visibility of all vertices is a desirable property in a phylogenetic *X*-network.

### Theorem 4.5

Suppose $N=(V,A,\rho ,\phi )$ is a tree-child *X*-network. Then every vertex *v* is visible.

### Proof

This result is shown in Cardona et al. (2009), where instead of saying “*u* is visible since it is on every path from the root to $\phi (x)$” the authors say “*x* is a strict descendant of *u*.” $\square$

### Corollary 4.6

Let $N=(V,A,\rho ,\phi )$ be a DCTC *X*-network or a normal *X*-network. Every $v\in V$ is visible.

### Proof

Every DCTC *X*-network and every normal *X*-network is tree-child. $\square$

### Theorem 4.7

Suppose $N=(V,A,\rho ,\phi )$ is a DCTC *X*-network. Suppose $u\in V$. Either

1. *u* is a leaf $\phi (x)$ for some $x\in X$; or
2. $u=p(x)$ for some $x\in X$, and *u* is hybrid with out-degree 1; or
3. *u* has out-degree at least 2, and for each child *c* of *u*, $cl(c)\subsetneq cl(u)$.

### Proof

If $outdeg(u)=0$, then (1) occurs. If $outdeg(u)=1$, let *c* be the unique child of *u*. Then $cl(u)=cl(c)$. Since *N* is DC by Theorem 4.2, (2) occurs. Otherwise $outdeg(u)\ge 2$. If *c* is a child of *u*, since *N* is DC it follows $cl(c)\subsetneq cl(u)$. $\square$

The following facts about tree-child networks, proved in Cardona et al. (2009), necessarily apply to DCTC networks:

- Let *m* be the maximal in-degree of a hybrid vertex and *n* be the number of leaves. Then $\vert V\vert \le (m+2)(n-1)+1$.
- For each *X* there is a metric (called the $\mu$-*distance*) on the class of tree-child phylogenetic *X*-networks.

### Theorem 4.8

Let $N=(V,A,\rho ,\phi )$ be a DCTC *X*-network.

1. Suppose *u* and *v* satisfy $cl(v)\subseteq cl(u)$. Then either $u\le v$ or else $u=\phi (x)$ for some $x\in X$, $v=p(x)$, and *v* is hybrid with out-degree 1, so $cl(u)=cl(v)=\{x\}$.
2. Suppose $(u,v)\in A$. If there is *w* distinct from *u* and *v* such that $cl(v)\subsetneq cl(w) \subsetneq cl(u)$, then (*u*, *v*) is redundant.

### Proof

(1) By Theorem 4.1, there exists $x\in cl(v)$ such that there is a tree-child path from *v* to $\phi (x)$, given by $v=v_0, v_1, \ldots , v_k=\phi (x)$. By hypothesis $x\in cl(u)$ so there is a path from *u* to $\phi (x)$. By Theorem 4.1 either $u=v_i$ for some $i>0$ or else $u\le v$. If $u\le v$, we are done. If instead, $u=v_i$, then $cl(u)\subseteq cl(v)$, whence $cl(u)=cl(v)$. Since *N* is CD this means that $u=v$ unless $\{u,v\}=\{p(x),\phi (x)\}$ as claimed.

(2) By Part (1) there is a path from *u* to *w* and a path from *w* to *v*. Hence, (*u*, *v*) is redundant. $\square$

### Corollary 4.9

If *N* is a DCTC *X*-network and $cl(v)\subsetneq cl(u)$, then $u\le v$.

**Fig. 3:**
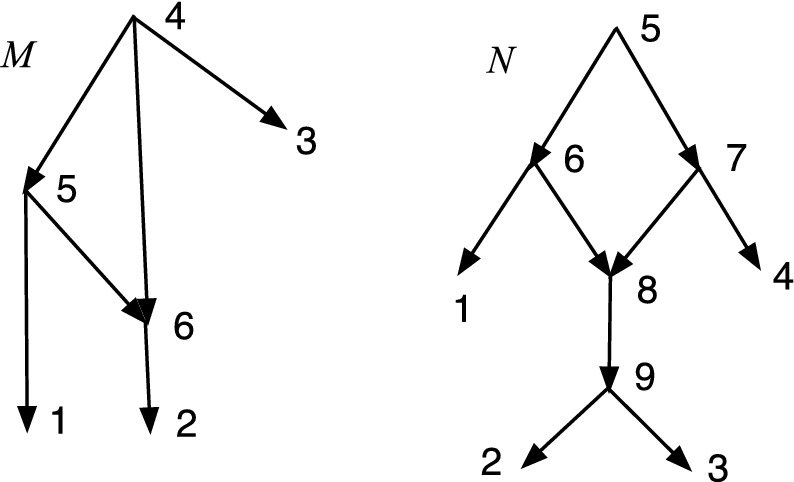
*M* is DCTC but not normal while *N* is normal but not DCTC

The property of being DCTC is closely related to being normal, but not the same. Figure 3 shows a network *M* that is DCTC but not normal, because (4, 6) is redundant. On the right *N* is normal but not DCTC because $cl(8)=cl(9)=\{2,3\}$. Nevertheless, we show below that given any DCTC *X*-network there is a closely related normal *X*-network.

**The operator S** If $N=(V,A,\rho ,\phi )$ is an *X*-network, let *S*(*N*) denote the result of contracting every arc (*u*, *w*) such that $outdeg(u)=1$. This applies even if *w* is a leaf. The operator *S* was briefly introduced in Willson (2022). Some basic properties are given in the next theorem.

### Theorem 4.10

Suppose $N=(V,A,\rho ,\phi )$ is an *X*-network.

1. $S(N)=(V',A', \rho ',\phi ')$ is an *X*-network except that a leaf may be hybrid.
2. *S*(*N*) contains no vertices with out-degree one.
3. If *N* is normal, then *S*(*N*) is both regular and normal.

Let $\psi :N\rightarrow S(N)$ be the projection map.

(4) $\psi$ is a CSD map but need not be leaf-preserving.
(5) For all $$u\in V$$, $$cl(u)=cl(\psi (u))$$.
(6) $$Cl(S(N))=Cl(N)$$.

### Proof

(1) follows naturally, with leaves possibly being hybrid because *D* may contain *X*-arcs. (2) is immediate by the construction. (3) follows from Willson (2010), and (4) is immediate.

For (5), if $(u,w)\in A$ and $outdeg(u)=1$, then $\psi (u)=\psi (w)=[u,w]$. Note $cl(u;N)=cl(w;N)$ since $cl(w)\subseteq cl(u)$ because of the arc (*u*, *w*), and every nontrivial path starting at *u* must pass through *w* since $outdeg(u)=1$. Hence $cl(u) = cl([u,w]) = cl(\psi (u))$. Now (6) is immediate since $\psi$ as a map of vertices is surjective. $\square$

If $N=(V,A,\rho ,\phi )$ is an acyclic *X*-network, let *R*(*N*) denote the result of removing all redundant arcs from *N*. More explicitly, let $A'$ be the set of arcs in *A* that are not redundant in *N*; then $R(N)=(V,A',\rho ,\phi )$. It is clearly an acyclic *X*-network. For more details, see Willson (2022).

An important use of *S* will be to compute *S*(*R*(*N*)) where *N* is an acyclic *X*-network. Note that *R*(*N*) then has no redundant arcs, and often the result can be simplified. The simplification might be performed by the operator *S*. If *N* is DCTC, we show below that *S*(*R*(*N*)) is normal.

**Fig. 4:**
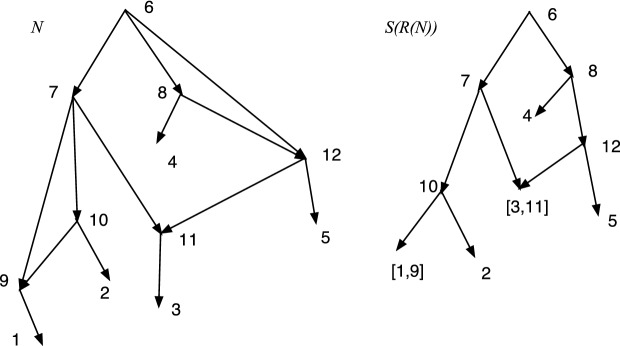
A DCTC *X*-network *N* with redundant arcs (7,9) and (6,12); and the normal network *S*(*R*(*N*))

Figure 4 gives an example of the computation of *S*(*R*(*N*)). Start with the DCTC *X*-network $N=(V,A,\rho ,\phi )$ on the left. The two redundant arcs are perfectly good arcs in *N*. Remove the two redundant arcs from *A* to form $A''$, so $R(N)=(V,A'', \rho ,\phi )$. The result has vertices 9 and 11 with out-degree one. Compute $S(R(N))=(V',A',\rho ',\phi ')$ by identifying each such with its unique child, forming new vertices [1,9] and [3,11] in $V'$. Note that in *S*(*R*(*N*)) the leaf [3,11] is hybrid. In *S*(*R*(*N*)) the map $\phi ': X\rightarrow V$ is given by $\phi '(1)=[1,9]$, $\phi '(2)=2$, $\phi '(3)=[3,11]$, $\phi '(4)=4$, $\phi '(5)= 5$.

The following theorem shows that if *N* is a DCTC *X*-network, then *S*(*R*(*N*)) is a normal network. This relationship shows a close relationship between DCTC networks and normal networks. It will be the basis for an expanded analysis in Sect. 6.

### Theorem 4.11

Suppose $N=(V,A,\rho ,\phi )$ is a DCTC *X*-network. Then

1. *R*(*N*) is a normal *X*-network.
2. For each vertex *u*, $cl(u;N)=cl(u;R(N))$.
3. Any arc (*u*, *w*) in *R*(*N*) either satisfies $cl(w)\subsetneq cl(u)$ or else there exists $x\in X$ such that $v=\phi (x)$, $u=p(x)$, and *p*(*x*) is hybrid with out-degree one.
4. *S*(*R*(*N*)) is a normal *X*-network, possibly having some leaves that are hybrid, and containing no vertex with out-degree one. No two vertices have the same cluster.

### Proof

(1) It is immediate that *R*(*N*) contains no redundant arcs, so we must only show *R*(*N*) is TC. Since *N* is tree-child, each vertex *u* that is not a leaf has a tree-child *w*. The arc (*u*, *w*) cannot be redundant. Otherwise, if (*u*, *w*) is redundant in *N*, then by redundancy there is a lengthening path starting at *u* and ending at *w* but not including the arc (*u*, *w*). Hence, $indeg(w)\ge 2$, a contradiction. It follows that (*u*, *w*) remains an arc in *R*(*N*), so *w* is a tree-child of *u* in *R*(*N*).

(2) Note that $x\in cl(u;N)$ iff there is a path in *N* from *u* to $\phi (x)$. A path from *u* to $\phi (x)$ of maximal length consists of only non-redundant arcs and remains a path in *R*(*N*). Hence, $cl(u;N)\subseteq cl(u;R(N))$. But trivially every path from *u* to $\phi (x)$ in *R*(*N*) is also such a path in *N*, proving Part (2).

(3) If (*u*, *w*) is an arc of *R*(*N*) then $cl(w;R(N))\subseteq cl(u;R(N))$. If $cl(w;R(N))=cl(u;R(N))$, then $cl(w;N)=cl(u;N)$ by Part (2). Since *N* is DC, $u=p(x)$ for some $x\in X$ and $w=\phi (x)$. This proves Part (3).

Since *R*(*N*) is normal, Part (4) follows from Theorem 4.10. $\square$

## Counting Vertices in DCTC *X*-Networks

In this section, we prove that the number of vertices in a DCTC *X*-network with *n* leaves is quadratic in *n*. We also study when a vertex is the $\text {mrca}(x,y)$ for distinct $x,y\in X$.

Call $x\in X$ *post-hybrid* if *p*(*x*) is hybrid with out-degree one. Equivalently, *x* is post-hybrid iff $cl(p(x))=\{x\}$. If *N* is a DCTC *X*-network, let ${\beta }(N)$ (or ${\beta }$ if *N* is understood) denote the number of $x\in X$ that are post-hybrid. Equivalently, ${\beta }(N)$ is the number of leaves whose parent is hybrid with out-degree one.

**Fig. 5:**
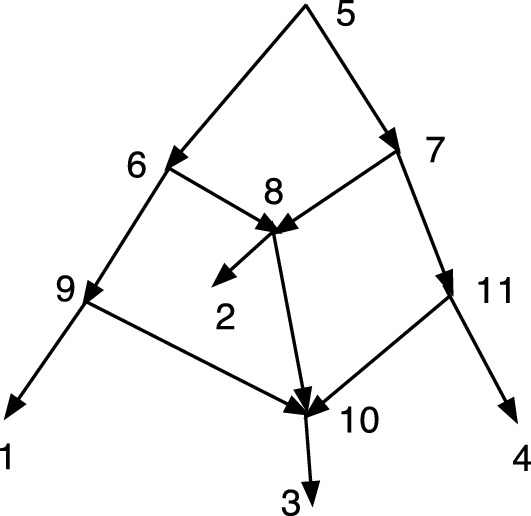
A DCTC *X*-network *N* with $n=4$ and ${\beta }(N)=1$

Figure 5 shows a DCTC *X*-network *N* with ${\beta }=1$, from the single post-hybrid leaf 3 since $10=p(3)$ has out-degree one. Note that 2 is not post-hybrid even though $8=p(2)$ is hybrid, since $outdeg(8)=2$.

### Theorem 5.1

Suppose $N=(V,A,\rho , \phi )$ is a DCTC *X*-network and $\vert X\vert = n$. Let $v(N)=\vert V\vert$ be the number of vertices, and $c(N)=\vert Cl(N) \vert$ be the number of distinct clusters of *N*. Then

1. $$v(N) = c(N)+{\beta }(N)$$.
2. $$c(N) \le n(n+1)/2$$.
3. $$v(N) \le n(n+1)/2 + {\beta }(N)$$.
4. $${\beta }(N) \le n-1$$.
5. $$v(N) \le n(n+3)/2-1$$.

### Proof

(1) Since *N* is CD, there is exactly one vertex for each member of *Cl*(*N*) except for the vertices *p*(*x*) for $x\in X$ such that *p*(*x*) is hybrid with out-degree one. For such *x*, there are two vertices *p*(*x*) and $\phi (x)$ with the same cluster $\{x\}$. Hence, (1) holds.

(2) Remove all redundant arcs of *N* to obtain *R*(*N*). By definition, *R*(*N*) contains no redundant arcs. Clearly *R*(*N*) is tree-child since no arc $(u,w)\in A$ which is an arc to a tree-child *w* of *u* is redundant; otherwise there would be a lengthening path from *u* to *w* which does not contain (*u*, *w*), hence another parent of *w*. It follows that *R*(*N*) is normal.

Moreover, the removal of redundant arcs does not change *cl*(*u*) for any vertex *u*. Hence, *R*(*N*) remains CD. For every arc (*u*, *w*) such that *cl*(*w*) is not $\{x\}$ for any $x\in X$, we must have $outdeg(u) > 1$; otherwise $cl(u)=cl(w)$, a contradiction. Suppress all the vertices of out-degree one identifying the ends of any arc (*u*, *w*) with $cl(u)=cl(w)=\{x\}$ for some $x\in X$. The result is *S*(*R*(*N*)) which will have exactly *c*(*N*) vertices, one for each cluster. But *S*(*R*(*N*)) is normal and has no vertices of out-degree one. By a result in Willson (2010), it has at most $n(n+1)/2$ vertices, proving Part (2). (The result stated in Willson (2010) differs slightly since, in that paper, *X* includes the root as well as the leaves.)

(3) follows immediately.

(4) Clearly ${\beta }(N) \le n$ since there are *n* leaves. Suppose ${\beta }(N)=n$. Then for every $x\in X$, *p*(*x*) is hybrid with out-degree 1. Yet *N* is tree-child and there must be a tree-path from the root $\rho$ to some leaf. Since every *p*(*x*) is hybrid, this is not possible. Hence, ${\beta }(N) < n$.

(5) $$v(N) = c(N)+{\beta }(N)\le n(n+1)/2 + (n-1) = n(n+3)/2-1$$. $$\square $$

Figure 5 shows a network *N* with $n=4$ leaves, $v(N)=11$ vertices, and ${\beta }(N)=1$. The inequality (3) of Theorem 5.1 is an equality for *N*. On the other hand, Part (5) will be improved in Theorem 5.6.

If *N* is tree-child, then it is immediate that for any vertex *u* there is a tree-child path from *u* to a leaf.

The notion of the *most recent common ancestor* $\text {mrca}(x,y)$ of two leaves $x,y\in X$ is defined in Sect. 2. It need not exist in general, but when it does it can provide useful information. Traits shared by species *x* and *y* may sometimes be traced back to $\text {mrca}(x,y)$ or earlier. It is therefore useful to know when $\text {mrca}(x,y)$ exists.

The following theorem shows in a DCTC *X*-network that every vertex with out-degree at least two has the form $\text {mrca}(x,y)$ for distinct $x,y\in X$. This result is interesting for its own sake as well as improving the upper bound given in Theorem 5.1(5) for the number of vertices.

### Theorem 5.2

Suppose $N=(V,A,\rho ,\phi )$ is a DCTC *X*-network. Suppose $u\in V$ has out-degree at least two. Let its distinct children be $c_1, c_2, \ldots , c_m$.

1. There exists $i\ne 1$ such that $cl(c_i)\nsubseteq cl(c_1)$.
2. For every $i=1,\dots ,m$ there exists *j* such that $cl(c_j)\nsubseteq cl(c_i)$.

Assume $c_1$ is a tree-child of *u*. Assume $cl(c_2)\nsubseteq cl(c_1)$. Let $u=u_0, u_1=c_1, \ldots , u_k=\phi (x)$ be a tree-child path from *u* to $\phi (x)$ through $c_1$, and let $c_2=v_0, v_1, \ldots , v_j=\phi (y)$ be a tree-child path from $c_2$ to $\phi (y)$. Then

(3) $$x\ne y$$ and $$\{x,y\} \subseteq cl(u)$$.
(4) If $w\in V$ satisfies $\{x,y\}\subseteq cl(w)$, then $w\le u$.
(5) $$u=\text {mrca}(x,y)$$.

### Proof

(1) Assume for each *i* that $cl(c_i)\subseteq cl(c_1)$. Then $cl(u) = cl(c_1) \cup \cdots \cup cl(c_m)$ $\subseteq cl(c_1) \cup \cdots \cup cl(c_1) = cl(c_1)$. But trivially $cl(c_1) \subseteq cl(u)$. Hence $cl(u) = cl(c_1)$, contradicting DC unless *u* is hybrid with out-degree one. But the latter contradicts that *u* has out-degree at least 2. Hence Part (1) is true.

Part (2) follows since in the proof of Part (1) the choice of $c_1$ was arbitrary.

The second half of (3) is immediate since $c_2$ is a child of *u*.

Next I claim that there are no *i* and *s* such that $u_i=v_s$. Otherwise suppose $u_i=v_s$ and *i* is minimal satisfying this condition. If $i=0$, then there is a cycle *u* to $c_2$ to $v_s=u$, a contradiction. If $i=1$ and $s\ge 1$, then $c_1$ has the parents *u* and $v_{s-1}$ which are distinct by the choice of *i*, contradicting that $c_1$ is a tree-child of *u*. If $i=1$ and $s=0$, then $c_1=c_2$, contrary to hypothesis. Thus, $i>1$. If $s\ge 1$, then $u_i$ has the parents $u_{i-1}$ and $v_{s-1}$ which are distinct by choice of *i*, contradicting that $u_i$ is a tree-child. Thus $u_i=v_0=c_2$, so $u_i$ has the parents $u_{i-1}$ and *u*, which is not possible since $i>1$. This proves the claim. This also proves that $x\ne y$, so (3) is true.

For (4) suppose $w\in V$ satisfies $\{x,y\}\subseteq cl(w)$. By Theorem 4.1 either $w = u_i$ for some *i*, $1\le i \le k$, or else $w\le u$. If $w\le u$, then (4) is true. Suppose instead $w=u_i$ for some *i*, $1\le i \le k$ (so $c_1\le w$). By Theorem 4.1 either $w=v_s$ for some *s* satisfying $1\le s \le j$, or else $w\le c_2$.

Consider the case where $w=v_s$. Then *w* has the parents $u_{i-1}$ and $v_{s-1}$, contradicting that $w=u_i$ is a tree-child. The remaining possibility is $w\le c_2$. Hence, $c_1\le w \le c_2$ so $cl(c_2)\subseteq cl(c_1)$. This contradicts the choice of $c_2$, proving Part (4). Then Part (5) follows from Part (4). $\square$

Note in the proof that *x* cannot be post-hybrid, but it is possible that $c_2=p(y)$ and *y* is post-hybrid.

### Remark

**Fig. 6:**
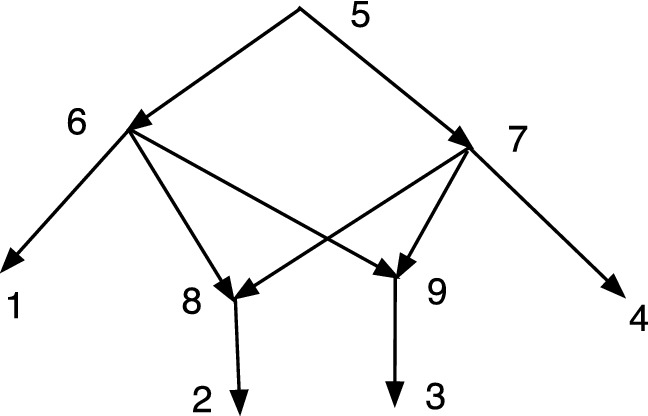
A DCTC *X*-network *N* in which $\text {mrca}(2,3)$ does not exist. Note also that 6 is not visible to 2 or to 3, and 7 is not visible to 2 or to 3

While Theorem 5.2 says that in a DCTC network many vertices have the form $\text {mrca}(x,y)$ for leaves *x*, *y*, it does not say that for all $x,y\in X$ that $\text {mrca}(x,y)$ exists. In Fig. 6, vertices 5, 6, and 7 are all the common ancestors of 2 and 3. We show that $\text {mrca}(2,3)$ does not exist. By the definition in Sect. 2, if *u* is a common ancestor of 2 and 3 and $u=\text {mrca}(2,3)$, then for every other common ancestor *v* of 2 and 3 we must have $v\le u$. But $u\ne 5$ since it is false that $6\le 5$; moreover, $u\ne 6$ since it is false that $7\le 6$; and $u\ne 7$ since it is false that $6\le 7$. Hence $\text {mrca}(2,3)$ does not exist. Briefly, 6 and 7 are both common ancestors of 2 and 3 as recent as possible in *N*, but neither is an ancestor of the other.

A related observation in Fig. 6 is that vertex 6 is not visible to either 2 or 3. It is not visible to 2 since there is the path 5, 7, 8, 2 from the root 5 to 2 that misses 6; it is not visible to 3 because there is the path 5, 7, 9, 3 that misses 6. But 6 is visible to 1 since every path from 5 to 1 includes vertex 6.

For Fig. 6, the proof of Theorem 5.2 merely says that $6=\text {mrca}(1,2)$ and $7=\text {mrca}(3,4)$. Note that the network of Fig. 6 is both normal and DCTC.

### Corollary 5.3

Suppose $N=(V,A,\rho ,\phi )$ is a DCTC *X*-network.

1. If $u\in V$ satisfies $outdeg(u)\ge 2$, then there exist distinct $x,y\in X$ such that $u=\text {mrca}(x,y)$. Moreover, at least one of *x* and *y* is not post-hybrid.
2. If $u\in V$ satisfies $outdeg(u)=1$, then there exists $x\in X$ such that $u=p(x)$ and *u* is hybrid.
3. The only vertices which do not have the form $\text {mrca}(x,y)$ for distinct $x,y\in X$ are the leaves $\phi (x)$ and the vertices *p*(*x*) that are hybrid with out-degree one, both having cluster $\{x\}$.

### Proof

(1) follows from the theorem. If *u* has out-degree one, let *c* be its unique child. Then $cl(u)=cl(c)$. Since *N* is DC, there exists $x\in X$ such that $u=p(x)$, $c=\phi (x)$, and *u* is hybrid, proving Part (2). Then Part (3) is immediate. $\square$

If *N* is DCTC, we also have the following result involving $\text {mrca}(B)$ where $B\subseteq X$. Every vertex *u* satisfies $u=\text {mrca}(cl(u))$ with the exception of the vertices $u=p(x)$ which are hybrid with out-degree one.

### Corollary 5.4

Let $N=(V,A,\rho ,\phi )$ be a DCTC *X*-network.

1. For each $u\in V$ such that $outdeg(u)\ge 2$, $u=\text {mrca}(cl(u))$.
2. If $outdeg(u)=1$, then there exists $x\in X$ such that $u=p(x)$ is hybrid with out-degree one, and $cl(u)=\{x\}$. In this case, $\text {mrca}(cl(u))=\phi (x)$.

### Proof

For (1), it is immediate that for $x\in cl(u)$, $u\le \phi (x)$. Thus *u* is a common ancestor of *cl*(*u*). Conversely, if $w \le \phi (x)$ for all $x\in cl(u)$, then in particular, there are $y,z\in X$ such that $u=\text {mrca}(y,z)$ by Corollary 5.3, and we have $w \le y$ and $w\le z$. Hence, $w\le u$. Thus, $u=\text {mrca}(cl(u))$.

For (2), if $outdeg(u)=1$, then by Corollary 5.3 there exists $x\in X$ such that $u=p(x)$ and *u* is hybrid. Thus, $cl(u)=\{x\}$, but clearly $\text {mrca}(x)=\phi (x)$ since $p(x)<\phi (x)$. $\square$

In Fig. 6, note that $cl(6)=\{1,2,3\}$. From Corollary 5.4, $6=\text {mrca}(cl(6))=\text {mrca}(1,2,3)$ as well as $6=\text {mrca}(1,2)$. But $\text {mrca}(cl(8))=\text {mrca}(\{2\})=2$.

The next result makes use of most recent common ancestors to find an upper bound on $\vert V\vert$.

### Theorem 5.5

Suppose $N=(V,A,\rho ,\phi )$ is a DCTC *X*-network, $n=\vert X\vert$, and ${\beta }$ is the number of post-hybrid leaves. Let $v(N)=\vert V\vert$. Then

$$v(N)\le (n^2 - {\beta }^2 + 3 {\beta } + n)/2 $$.

### Proof

Let $V_2$ denote the set of vertices with out-degree at least 2. Let *P* denote the set of post-hybrid members of *X*, so $\vert P \vert ={\beta }$.

If $x\in P$, then every path to $\phi (x)$ from another vertex includes the hybrid vertex *p*(*x*). It follows that there is no tree-child path from $u\in V_2$ to $\phi (x)$ for $x\in P$, only from *p*(*x*) to $\phi (x)$.

For each vertex $u\in V_2$, we first choose a tree-child $c_1$ leading to a tree-child path from *u* to $\phi (x)$. From another child $c_2$ of *u* (such that it is false that $cl(c_2)\subseteq cl(c_1)$), we obtain a tree-child path to $\phi (y)$, obtaining an *allowed* 2-set $\{x,y\}$. Note that *x* can’t be in *P*. If $c_2$ has out-degree one, then $c_2 =p(y)$ and $y\in P$; but otherwise *y* is not in *P*.

The number of 2-sets $\{x,y\}$ with no member of *P* is $$\left( {\begin{array}{c}n-{\beta }\\ 2\end{array}}\right) $$. The number of 2-sets with exactly one member of *P* is ${\beta } (n-{\beta })$. Hence, the number of allowed 2-sets is $$\left( {\begin{array}{c}n-{\beta }\\ 2\end{array}}\right) + {\beta } (n-{\beta })$$ so $$\vert V_2 \vert \le \left( {\begin{array}{c}n-{\beta }\\ 2\end{array}}\right) + {\beta } (n-{\beta })$$.

The number of vertices of out-degree 1 is $\vert P \vert = {\beta }$, and the number of leaves is *n*. Hence, the number of vertices is $\vert V \vert = \vert V_2 \vert + \vert P \vert + n$ $$\le \left( {\begin{array}{c}n-{\beta }\\ 2\end{array}}\right) + {\beta } (n-{\beta }) + {\beta } + n$$ $= (n^2 - {\beta }^2 + 3 {\beta } + n)/2$. $\square$

We now obtain our best upper bound for *v*(*N*):

### Theorem 5.6

Suppose $N=(V,A,\rho ,\phi )$ is a DCTC *X*-network and $n=\vert X\vert$. Then $v(N) \le (n^2 + n + 2)/2$.

### Proof

For fixed *n* the function $f({\beta })= (n^2 - {\beta }^2 + 3 {\beta } + n)/2$ has a maximum value when $f'({\beta }) = -2 {\beta } + 3 = 0$ or ${\beta }=3/2$. We then obtain that $v(N) \le f(3/2) = (n^2 - (3/2)^2 + 3(3/2) + n)/2 = (4 n^2 + 4 n + 9)/8 = (n^2 + n + 9/4)/2$. Since *v*(*N*) is an integer, $v(N)\le (n^2+n+2)/2$. $\square$

For the DCTC *X*-network in Fig. 5, $n=4$ and there are 11 vertices, so the upper bound of Theorem 5.6 is tight in this example.

We show in Theorem 7.1 that the upper bound in Theorem 5.6 is tight for $n\ge 3$.

**Fig. 7:**
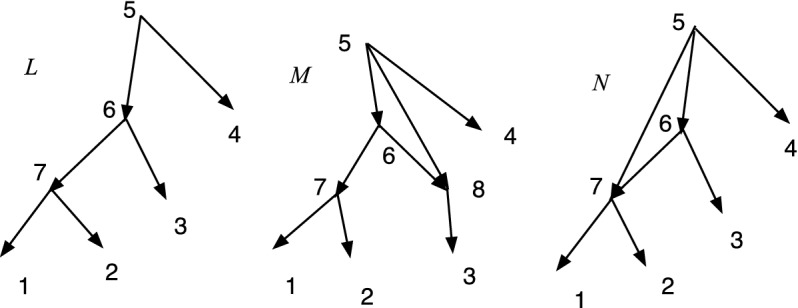
Three DCTC *X*-networks *L*, *M*, and *N* with $Cl(L)=Cl(M)=Cl(N)$. Hence $d_{RF}(L,M)=d_{RF}(L,N)=d_{RF}(M,N) = 0$

## DCTC Networks with the Same Clusters

Several different DCTC *X*-networks $N_1$, $N_2, \cdots$, $N_k$ can all satisfy that

$d_{RF}(N_i,N_j)=0$, so they have the same clusters. In this section, we study their relationship. Our main theorem for this section states that they all have the same $S(R(N_i))$.

Their differences will involve redundant arcs. For example, consider Fig. 7, where $X=\{1,2,3,4\}$. In *L*, $p(3)=6$ while in *M*, $p(3)=8$; the extra vertex 8 in *M* allows the redundant arc (5, 8). The redundant arc (5,7) in *N* did not require a new vertex. In general, adding a redundant arc between two existing vertices in a DCTC *X*-network *N* to create *M* does not change the set of clusters and hence yields $d_{RF}(M,N)=0$. One must take care that the result remains tree-child.

Suppose *X* is a nonempty set. Let ${\mathcal {C}}$ be a collection of nonempty subsets of *X* such that for each $x\in X$, $\{x\} \in {\mathcal {C}}$ and $X\in {\mathcal {C}}$. Baroni et al. in Baroni et al. (2004) construct an *X*-network which we shall denote $Reg({\mathcal {C}})$. The vertex set will be the set ${\mathcal {C}}$, and $Reg({\mathcal {C}})$ is the cover digraph of ${\mathcal {C}}$. More explicitly $Reg({\mathcal {C}})=({\mathcal {C}}, A, X, \phi )$ where there is an arc $(C_1,C_2)\in A$ iff (a) $C_2 \subsetneq C_1$, and (b) there is no $C_3\in {\mathcal {C}}$ distinct from $C_1$ and $C_2$ such that $C_1\subsetneq C_3\subsetneq C_2$. The root is *X*, and the map $\phi :X\rightarrow {\mathcal {C}}$ is $\phi (x)=\{x\}$.

Following are some properties of $Reg({\mathcal {C}})$ from Baroni et al. (2004):

1. $Reg({\mathcal {C}})$ is a regular *X*-network (possibly having hybrid leaves).
2. An *X*-network *N* (possibly having hybrid leaves) with cluster set *Cl*(*N*) is regular iff *N* is isomorphic with *Reg*(*Cl*(*N*)).
3. $Reg({\mathcal {C}})$ contains no redundant arcs.

**Fig. 8:**
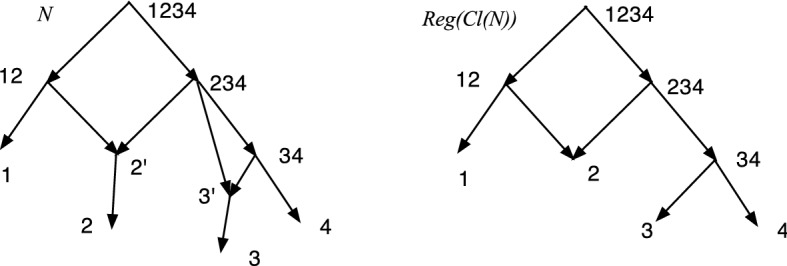
A DCTC *X*-network *N* and $Reg({\mathcal {C}})$ for ${\mathcal {C}}=Cl(N)= \{\{1\}, \{2\}, \{3\}, \{4\}$, $\{1,2,3,4\}$, $\{1,2\}, \{3,4\}$, $\{2,3,4\} \}$. The vertices are labeled by their clusters. In *N*, $2'$ and $3'$ label parents of 2 and 3 with out-degree one. Note the hybrid leaf 2 in $Reg({\mathcal {C}})$

Figure 8 displays $Reg({\mathcal {C}})$ where ${\mathcal {C}}= \{\{1\}, \{2\}, \{3\}, \{4\}, \{1,2,3,4\}$, $\{1,2\}$, $\{3,4\}, \{2,3,4\} \}$.

### Theorem 6.1

Suppose $N=(V,A,\rho ,\phi )$ is a DCTC *X*-network. Then

1. *S*(*R*(*N*)) is *X*-isomorphic with *Reg*(*Cl*(*N*)).
2. Suppose $N_1$ and $N_2$ are DCTC *X*-networks and $d_{RF}(N_1,N_2)=0$. Then $S(R(N_1))$ and $S(R(N_2))$ are *X*-isomorphic.

### Proof

(1) By Theorem 4.11*S*(*R*(*N*)) is normal and contains no vertices with out-degree one. By results in Willson (2010), this network is therefore regular. From Baroni et al. (2004) we know that *S*(*R*(*N*)) is *X*-isomorphic with *Reg*(*Cl*(*S*(*R*(*N*)))). But *Cl*(*S*(*R*(*N*))) $=Cl(R(N))$ by Theorem 4.10, while $Cl(R(N))=Cl(N)$ by results in Willson (2022), proving Part (1).

(2) From Part (1), for $i=1,2$, $S(R(N_i))$ is *X*-isomorphic with $Reg(Cl(N_i))$. But by hypothesis, $Cl(N_1)=Cl(N_2)$. The result follows. $\square$

Theorem 6.1(2) implies that, given a DCTC *X*-network *N*, all DCTC *X*-networks *M* such that $d_{RF}(N,M)=0$ can be found as follows: Compute $M_0=S(R(N))=Reg(Cl(N))=(V,A,\rho ,\phi )$. If there is any leaf *u* of *M* that is hybrid, then there exists a unique $x\in X$ such that $u=\phi (x)$. For all such $\{x\}$, modify $M_0$ by adding a new arc $(u,\phi (x))$, making $u=p(x)$ and producing a new network $M_1$. Now $M_1$ is a DCTC *X*-network. Next recursively adjoin redundant arcs to $M_1$, taking care that the result will still be tree-child and DC. The redundant arcs can be of form (*a*, *b*) where *a* and *b* are vertices of $M_1$. Alternatively, they can be of form (*a*, *b*) where *a* is a vertex of $M_1$ and *b* is a new vertex in the middle of what was the arc $(p(x),\phi (x))$, where $outdeg(p(x))>1$. Any network $M_k$ so obtained will satisfy $d_{RF}(M_k,N)=0$.

For example, consider Fig. 8 again. Given *N*, we compute *Cl*(*N*) and then *Reg*(*Cl*(*N*)). Suppose we want to reconstruct *N*. Let $M_0=Reg(Cl(N))$. Then $M_1$ replaces 2 by $2'$ and adds the arc $(2',2)$. Next we subdivide (34, 3) at $3'$ and adjoin the redundant arc $(234,3')$. At this stage, we have reconstructed *N*. We could continue to get another DCTC *X*-network with the same clusters as *N* by adjoining new redundant arcs $(1234,2')$ and/or $(1234,3')$. We could not, however, adjoin a new redundant arc (1234, 34) since then all children of 234 would be hybrid and the result would not be tree-child.

## The Upper Bound on the Number of Vertices is Tight

Theorem 5.6 asserted that if *N* is a DCTC *X*-network with *n* leaves, then the number of vertices is at most $(n^2+n+2)/2$. In this section we show that this upper bound is tight. We shall construct a sequence of DCTC networks $L_n$, for $n\ge 3$, where $L_n$ has *n* leaves and $v_n=(n^2+n+2)/2$ vertices. It turns out that $L_n$ contains no redundant arcs, hence is also normal. The construction mimics a construction in Bickner (2012) of interesting normal networks. The construction will be inductive.

**Fig. 9:**
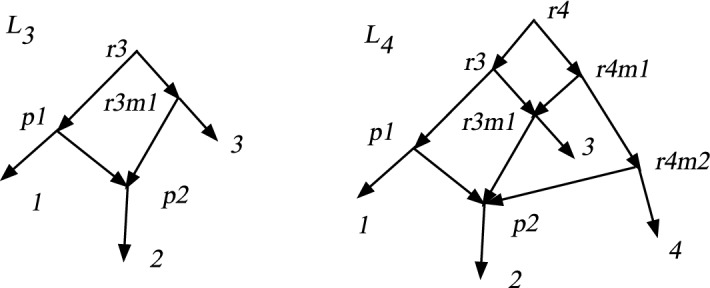
The DCTC networks $L_3$ and $L_4$. $L_n$ has $v_n=(n^2+n+2)/2$ vertices and *n* leaves

**Fig. 10:**
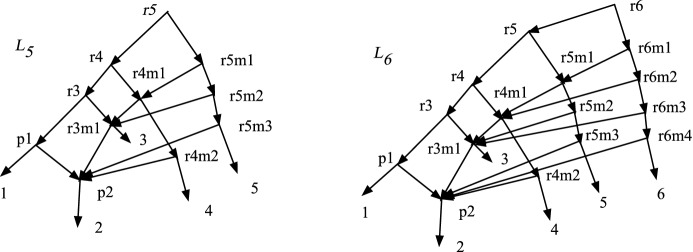
The DCTC networks $L_5$ and $L_6$. $L_n$ has $v_n=(n^2+n+2)/2$ vertices and *n* leaves

We start with $L_3$ shown in Fig. 9 left with 3 leaves. It is easily seen to be DCTC and normal and has 7 vertices; note that $v_3=7$. Also note that 2 is post-hybrid and $p(2)=p2$ is the only hybrid vertex. The root is *r*3 and the child *r*3*m*1 is the child of *r*3 that is an ancestor of 3.

To obtain $L_4$, shown in Fig. 9 right, we add 4 new vertices to $L_3$; these are a new root *r*4 with a tree-child path of new vertices *r*4, *r*4*m*1, *r*4*m*2, 4. Note *r*4 also has tree-child *r*3; *r*4*m*1 also has child *r*3*m*1, and *r*4*m*2 also has child *p*2. Note that *r*3*m*1 has become hybrid with tree-child 3, while *r*3 still has the tree child *p*1. Since *p*2 was already hybrid in $L_3$, *r*3*m*1 is the only new hybrid. Every non-leaf vertex of $L_3$ still has a tree-child in $L_4$. Thus $L_4$ is TC. For every vertex *v* of $L_3$, $cl(v;L_4)=cl(v;L_3)$. The new vertices have clusters $cl(r4;L_4)=cl(r3;L_3)\cup \{4\}$, $cl(r4m1;L_4)=cl(r3m1;L_3)\cup \{4\}$, $cl(r4m2;L_4)=cl(p2;L_3)\cup \{4\}$. Hence $L_4$ is DC. Finally, $L_4$ has 7+4 = 11 vertices, and $v_4=11$. Since it has no redundant arcs, it is also normal.

Given $$L_n$$, we show how to define $$L_{n+1}$$. The process is illustrated in Fig. 10 by showing $$L_5$$ and $$L_6$$. We modify $$L_n$$ by letting $$n'=n+1$$ and adding $$n'$$ new vertices along a tree-child path $$rn', rn'm1, r n'm2, \ldots , r n'm(n'-2), n'$$, hence with new arcs $$(r n', r n'm1)$$, $$(r n'm1, r n'm2),\ldots $$, $$(r n'm(n'-2),n')$$. We also add arcs $$(r n', r n)$$, $$(r n'm1, r nm1)$$, $$(r n'm2, r(n-1)m1)$$, $$ (r n'm3, r(n-2)m1)$$, $$\ldots , (r n'm(n'-2), p2)$$. The only new hybrid vertex is *rnm*1 but *rn* still has the tree-child *rn*. All non-leaf vertices of $$L_n$$ still have a tree-child; and the new vertices of $$L_{n+1}$$ have a tree-child from the tree-child path, so $$L_{n+1}$$ is TC. Since it has no redundant arcs, it is also normal.

For each vertex *v* of $L_n$, we have $cl(v;L_{n+1})=cl(v;L_n)$. The new vertices satisfy $cl(r n';L_{n+1})=cl(r n;L_n)\cup \{n+1\}$, $cl(r n'm1;L_{n+1})= cl(r nm1; L_n)\cup \{n+1\}$, $\ldots , cl(r n'm(n'-2); L_{n+1})= cl(p2;L_n)\cup \{n+1\}$. It is easy to see therefore that $L_{n+1}$ is DC hence DCTC. Finally, since $L_n$ had $v_n$ vertices, $L_{n+1}$ has $v_n+n+1$ vertices, which equals $(n^2+n+2)/2+(n+1) = v_{n+1}$ by algebra.

We have proved the following:

### Theorem 7.1

For $n\ge 3$, there exists a network with *n* leaves which is both DCTC and normal and which has $v_n=(n^2+n+2)/2$ vertices. Hence, the upper bound in Theorem 5.6 is tight for all $n\ge 3$.

If $n=2$ a DCTC *X*-network with *n* leaves has at most 3 vertices, where $3<4=v_2$, so the restriction on *n* is needed.

## DCTC Networks from Normal Networks

Recall from Theorem 3.5 that when *N* is an acyclic *X*-network there is an *X*-network $\text {SCD}(N)$ which is successively-cluster-distinct (SCD) with other interesting properties. In this section, we see that, given a normal network *N*, it follows that $\text {SCD}(N)$ is a DCTC network.

### Theorem 8.1

Let $N=(V,A,\rho ,\phi )$ be a normal *X*-network. Then $\text {SCD}(N)$ is a DCTC *X*-network containing no redundant arcs.

### Proof

From Willson (2022), the first step to form $\text {SCD}(N)$ is to let $D=\{(u,v)\in A: cl(u;N)=cl(v;N)$ and *v* is not a leaf$\}$ and compute $M_D(N)$.

If $(u,v)\in D$ and $outdeg(u)=1$, then (*u*, *v*) is contracted in the formation of $\text {SCD}(N)$.

If $(u,v)\in A$ and $outdeg(u)>1$, I claim $cl(u)\ne cl(v)$. To see this, let *w* be another child of *u* beside *v*. Since *N* is normal, we may assume at least one of $\{v,w\}$ is a tree-child of *u*. Choose a tree-path from *v* to the leaf *x* and from *w* to the leaf *y*. Note that $u\le x$ and $u\le y$. From Willson (2010), $u=\text {mrca}(x,y)$. But since $cl(u)=cl(v)$ we have $v\le x$ and $v\le y$, whence $v\le u$ by the $\text {mrca}$ property. This is a contradiction, proving $cl(u)\ne cl(v)$. Hence $(u,v)\notin D$.

By Theorem 4.3 of Willson (2022) $M_D(N)$ is SCD, although possibly containing a trivial vertex of form *p*(*x*) for some $x\in X$. In this situation, let the unique parent of *p*(*x*) be denoted *u*(*x*).

Now Theorem 4.4 of Willson (2022) shows $\text {SCD}(N)$ is obtained by contracting each such arc (*u*(*x*), *p*(*x*)) to suppress the trivial vertex *p*(*x*). Thus, $\text {SCD}(N)$ is SCD.

But it is clearly tree-child as well since whenever we contracted $(u,v)\in D$ into a point [*u*, *v*] the tree-child of *v* becomes a tree-child for [*u*, *v*]. And whenever we contracted (*u*(*x*), *p*(*x*)) into [*u*(*x*), *p*(*x*)], $\phi (x)$ becomes a tree-child of [*u*(*x*), *p*(*x*)]. Hence $\text {SCD}(N)$ is DCTC by Corollary 4.3. Since *N* contained no redundant arcs, the same is true about $\text {SCD}(N)$. $\square$

**The operator** $S'$. The operator *S* is described in Sect. 4. Here we give a slight modification. Given a normal *X*-network *N*, let $S'(N)$ be defined by first contracting arcs in $D=\{(u,v)\in A: outdeg(u)=1$ and *v* is not a leaf$\}$ and then contracting any remaining arcs (*u*(*x*), *p*(*x*)) where, for $x\in X$, *p*(*x*) is a trivial vertex with unique parent *u*(*x*) having out-degree at least 2.

The proof of Theorem 8.1 shows that if *N* is normal, then $S'(N)$ is a simpler construction of $\text {SCD}(N)$.

### Corollary 8.2

Let $N=(V,A,\rho ,\phi )$ be a normal *X*-network. Then $S'(N)=\text {SCD}(N)$ and is a DCTC *X*-network, but contains no redundant arcs.

We will illustrate the use of Corollary 8.2 in Examples 1, 2 and 3.

## Finding a Standard DCTC *X*-Network from a Given *X*-Network

Given an *X*-network *N*, this section shows how to produce a uniquely determined *X*-network which is DCTC and which we denote $\text {DCTC}(N)$. The procedure resembles that in Willson (2022) used to produce a uniquely determined network $\text {Norm}(N)$ which is normal. While the procedure in Willson (2022) involves the removal of redundant arcs, the procedure in this section does not involve explicit removal of redundant arcs and consequently has some advantages.

Let $N=(V,A,\rho ,\phi )$ be an *X*-network. A vertex *v* of *N* is a *tree-child obstacle* or (in this section) an *obstacle* if

1. *v* is not a leaf; and
2. every child of *v* is hybrid. Hence, whenever (*v*, *u*) is an arc, *u* has a parent other than *v*.

An *X*-network *N* is *tree-child obstacle-free* if it contains no tree-child obstacle.

### Theorem 9.1

Suppose *N* is an *X*-network that is tree-child obstacle-free. Then *N* is a tree-child *X*-network.

### Proof

By hypothesis, for every vertex *v* that is not a leaf, there is an arc (*v*, *c*) with $indeg(c) = 1$. It follows that *c* is a tree-child of *v*. Hence, *N* is tree-child. $\square$

Given an *X*-network *N*, suppose we seek a related DCTC *X*-network. Our strategy will be to compute $\text {SCD}(N)$ to make it SCD. Then we recursively remove tree-child obstacles until there are no more obstacles. If we seek to obtain a uniquely determined tree-child network we are careful not to make arbitrary choices of which arcs to merge.

Just as for pre-normal obstacles in Willson (2022), there are different types of tree-child obstacles.

Let *N* be an *X*-network. Suppose *c* is a tree-child obstacle. An *allowable 1-fold parent chain of c* is a path $p^1, c$ where $p^1$ is a parent of *c*, $(p^1,c)$ is not redundant, and such that $p^1$ has a tree-child $d \ne c$. An obstacle *c* is of type 1 if *c* has an allowable 1-fold parent chain. If *c* has type 1, and $p^1, c$ is an allowable parent chain, let $Dc(p^1,c) = \{ (p^1,c)\}$. We will be merging the arc in $Dc(p^1,c)$.

Suppose *c* is a tree-child obstacle. An *allowable k-fold parent chain for c* is a path $p^k, p^{k-1}, \ldots , p^1, p^0=c$ such that no arc $(p^i, p^{i-1})$ is redundant, and $p^k$ has a tree-child $d \ne p^{k-1}$. An obstacle *c* is of *type* *k* if

- *c* is not of type $1, \ldots , k-1$;
- *c* has an allowable *k*-fold parent chain.

In this situation, for each such allowable *k*-fold parent chain write

$$\begin{aligned} Dc(p^k,p^{k-1}, \ldots , c)= \{(p^k,p^{k-1}), \ldots , (p^1, c)\}. \end{aligned}$$

We will be merging the arcs in $Dc(p^k,p^{k-1}, \ldots , c)$.

**Fig. 11:**
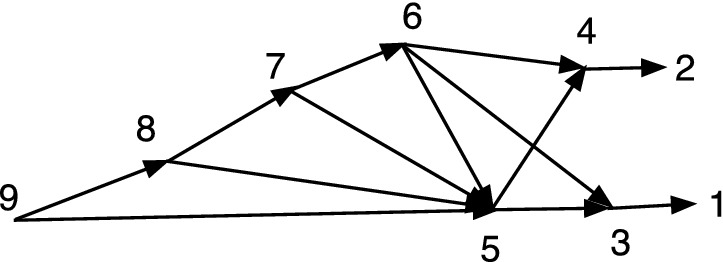
An *X*-network *N* in which tree-child obstacle 6 has no type

It is false that in every *X*-network every tree-child obstacle has a type. Figure 11 shows an *X*-network *N* in which 6 is a tree-child obstacle that has no type. Nevertheless, in an SCD *X*-network, the next result shows that every tree-child obstacle has a type.

### Theorem 9.2

Let *N* be an SCD *X*-network. Then every tree-child obstacle *c* has a unique type.

### Proof

It is clear that the type, if it exists, is unique. The root $\rho$ satisfies $cl(\rho ) = X$. Since *N* is *SCD*, every child *c* of $\rho$ satisfies $cl(c) \ne X$. For every $x \in X$, there is child *d* of $\rho$ satisfying $x \in cl(d)$. If we choose such a child *d* with maximal *cl*(*d*), then $(\rho ,d)$ cannot be redundant and *d* has no parent other than $\rho$, so *d* is a tree-child of $\rho$. Since *N* is SCD and no child of $\rho$ can have cluster *X*, $\rho$ has at least two tree-children.

Consider a path from $\rho$ to *c* which has maximal length *k*. Write it as $u_0=\rho , u_1,$ $\ldots , u_k = c$. By Lemma 2.1 of Willson (2022), every arc on this path is non-redundant. Let $p^i = u_{k-i}$, so this path is $\rho = p^k, p^{k-1}$, $\ldots , p^1, c$; it is an allowable *k*-fold parent chain of *c* since $\rho$ has a tree-child other than $p^{k-1}$. Hence, *c* has type $\le k$. $\square$

The next result shows a way to remove a single obstacle of type 1.

### Lemma 9.3

Suppose *N* is an *X*-network and *c* is a type 1 tree-child obstacle with allowable 1-fold parent chain *p*, *c* such that *p* has tree-child $d\ne c$. Let $D = \{(p,c)\}$. Then, in $M_D(N)$, $\psi (c) = [p,c]$ has the tree-child *d* and is not an obstacle.

### Proof

Since (*p*, *c*) is not redundant, *D* is strongly closed and from Willson (2022) $M_D(N)$ is obtained by merely merging the ends of the arc (*p*, *c*) into a vertex [*p*, *c*]. In $M_D(N)$, there is an arc ([*p*, *c*], *d*). If *q* is any parent of *d* in $M_D(N)$ other than [*p*, *c*], then *q* was a parent of *d* in *N* as well, contradicting that *d* was a tree-child of *p*. $\square$

The result generalizes to obstacles of type *k*, with a proof similar to that of Lemma 7.4 in Willson (2022).

### Lemma 9.4

Let *N* be an *X*-network with tree-child obstacle *c* of type *k*. Suppose $p^k, \ldots , c$ is an allowable *k*-fold parent chain, where $p^k$ has tree-child $d \ne p^{k-1}$. Let $D = Dc(p^k, \ldots , c)$ $= \{ (p^k,p^{k-1}), (p^{k-1}, p^{k-2}), \ldots , (p^1, c)\}$. Assume *D* is strongly closed. Form $M_D(N)$ and let $\psi :N \rightarrow M_D(N)$ be the projection. Then $\psi (c)= [p^k, p^{k-1}, \ldots , c]$ has tree-child *d*, so $\psi (c)$ is not an obstacle in $M_D(N)$.

The following lemma shows that, usually, once an obstacle is removed, it does not reappear when subsequent arcs are merged. Its proof is like that of Lemma 7.5 in Willson (2022).

### Lemma 9.5

Suppose (*p*, *c*) is a non-redundant arc in the *X*-network *N* and $N'$ is obtained by identifying *p* and *c*. Let $\psi : N \rightarrow N'$ be the projection. Suppose (*a*, *b*) is a non-redundant arc in *N* and *b* is a tree-child of *a*. Assume $b \ne p$, $b\ne c$. Then $(\psi (a),\psi (b))$ is a non-redundant arc of $N'$ and $\psi (b)$ is a tree-child of $\psi (a)$.

These results lead to a procedure for finding a DCTC network closely related to a given *X*-network *N*: The procedure is similar to that in Willson (2022) for finding $\text {Prenorm}(N)$.

**Procedure** DCTC.

**Input** An *X*-network *N*.

**Output** An *X*-network and an integer.

1. Let $$i=0$$ and $$N_0=N$$.

2. If $N_0$ is tree-child and SCD, go to step 9. Otherwise, go to step 3.

3. Let $$N_1 = \text {SCD}(N_0)$$ and $$i=1$$.

4. If $N_1$ is tree-child, go to step 9. Otherwise, go to step 5.

5. For each tree-child obstacle *c* in $N_i$

(5a). Compute the type *k*.

(5b). Initialize $$D(c) = \emptyset $$.

(5c). For each allowable *k*-fold parent chain $p^k, \ldots , c$ for *c*, let $Dc(p^k, \ldots , c) = \{(p^k, p^{k-1}), (p^{k-1},p^{k-2}), \ldots , (p^1,c)\}$.

(5d). Let $Dc=\cup \{Dc(p^k, \ldots , c)\}$ where the union is over all allowable *k*-fold parent chains for *c*.

6. Let $D = \cup Dc$ where the union is over all tree-child obstacles *c*.

7. Let $N_{i+1} = \text {SCD}(M_D(N_i))$. Set $i:= i+1$ so that the current network becomes $N_i$.

8. If $N_i$ has no obstacles, go to step 9. Otherwise, go to step 5.

9. Output $N_i$ and the integer *i*.

The network which is output from procedure DCTC applied to the *X*-network *N* will be denoted $\text {DCTC}(N)$. The integer output will be called the *height* of $\text {DCTC}(N)$. Note that the height is 0 only if *N* is itself DCTC, and the height is 1 only if $\text {SCD}(N)$ is DCTC.

### Theorem 9.6

Suppose we apply the procedure DCTC to an *X*-network $N=(V,A,\rho ,\phi )$.

1. The procedure terminates and outputs an integer *r* and an *X*-network $N_r$.
2. $N_r$ is DCTC and will be denoted $\text {DCTC}(N)$.
3. $\text {DCTC}(N)$ depends only on the structure of *N* and not any arbitrary choices.
4. There is a leaf-preserving CSD map $\psi : N\rightarrow \text {DCTC}(N)$.
5. Let $E_1 = \{(u,v)\in A: \psi (u)\ne \psi (v)\}$. Then $(\psi ^{-1},E_1)$ is a wired lift of $\text {DCTC}(N)$ into *N*.

### Proof

(1) By construction, for $i\ge 1$ $N_i$ is SCD. If $N_i$ has an obstacle *c*, then by Theorem 7.2 *c* has a type, so step (5a) can be carried out. Hence, the procedure is well-defined. Every time, the procedure enters step 5, the network has at least one obstacle, so the set *D* in step 6 is nonempty. Then at least one arc of $N_i$ is merged in the formation of $M_D(N_i)$, so $M_D(N_i)$ has fewer vertices than $N_i$. Since *N* is finite, it follows that the procedure terminates.

(2) It is immediate that the output $N_r$ is SCD since $N_r=\text {SCD}(M_D(N_{r-1}))$. Moreover, $N_r$ is tree-child by Theorem 7.1 since it has no tree-child obstacles. (Otherwise, the procedure would have computed $N_{r+1}$). Hence, it is DCTC.

(3) is immediate since no choices are made between different obstacles or allowable parent chains for an obstacle.

(4) is immediate if $r=0$ and follows from Theorem 3.5 if $r=1$. Assume $r>1$. Let $\psi _1:N\rightarrow \text {SCD}(N)=N_1$ be the projection. By Theorems 3.4 and 3.5, there is a leaf-preserving projection $\psi _2: N_1\rightarrow N_2=\text {SCD}(M_D(N_1))$. Similarly for $i\le r$, there is a CSD projection $\psi _i: N_{i-1}\rightarrow N_i$. Then the composition $\psi = \psi _{r-1} \circ \psi _{r-2} \circ \cdots , \circ \psi _2 \circ \psi _1$ is a leaf-preserving CSD map from *N* to $\text {DCTC}(N)$.

(5) $(\psi ^{-1},E_1)$ is a wired lift by Theorem 3.1. $\square$

Just as in Willson (2022), one can define a procedure VARIANT DCTC in which Step (5d) is omitted and Step (5c) is replaced by

(5c’). Select one allowable *k*-fold parent chain $p^k, \ldots , c$ for *c* and let $Dc=\{(p^k,p^{k-1}), (p^{k-1},p^{k-2}), \ldots , (p^1,c)\}$.

The network that is output may be denoted $\text {DCTC}_{V,C}(N)$ or $\text {DCTC}_V(N)$, where *C* indicates the choice of each parent chain when there are more than one possible. As in Willson (2022) $\text {DCTC}_{V,C}(N)$ will depend on the choice of the parent chains (when there are more than one). On the other hand, the result may have higher resolution than $\text {DCTC}(N)$ and may sometimes be useful.

Some detailed examples will be presented in Sect. 11.

## Some Parameters of Networks

In the examples in Sect. 11, we compare several different networks for the same collection *X* of leaves. We use the following numerical parameters in related tables. These parameters are chosen in part because they generalize parameters useful in analyzing phylogenetic trees. Our goal is in part to see which phylogenetic networks can be most useful for analyzing gene flow in complicated situations. Quantities useful in trees can sometimes be generalized in more than one way to networks. In some cases, we will compare the parameter for a network with the corresponding parameter for rooted trees.

Let $N=(V,A,\rho ,\phi )$ be an *X*-network.

- $n=\vert X\vert$ is the number of leaves.
- $v=\vert V\vert$ is the number of vertices.
- $a = \vert A\vert$ is the number of arcs.
- *h* is the number of hybrid vertices. For a tree, $h=0$. In a DCTC network, $h\le n-2$ by Theorem 4.4.
- *r* is the number of redundant arcs. For a tree or a normal network, $r=0$.
- *o*1 is the number of vertices with out-degree one. In a tree such vertices would also have in-degree one and hence would be suppressed as trivial. Hence for a tree, $o1=0$. For a DCTC network, $o1={\beta }(N)$.
- *o*2 is the number of vertices with out-degree 2 or higher. In a tree we expect all vertices other than leaves have out-degree 2 or higher, so $o2=v-n$. In general, $o2=v-n-o1$.
- *o*2*m* is the number of vertices with out-degree 2 or greater which equal $\text {mrca}(x,y)$ for some 2-set $\{x,y\}$ from *X*. In a tree, $o2m=o2$. By Theorem 5.2, in a DCTC network $o2m=o2$ and the same is true for normal networks (Willson 2010).
- *mrca* is the number of 2-sets of leaves $\{x,y\}\subseteq X$ for which $\text {mrca}(x,y)$ exists. There are $$\left( {\begin{array}{c}n\\ 2\end{array}}\right) $$ such 2-sets, and in a tree $$mrca=\left( {\begin{array}{c}n\\ 2\end{array}}\right) $$. It often happens that the same vertex $u=\text {mrca}(x,y)$ for several different $\{x,y\}$. A biologist might be interested in $\text {mrca}(x,y)$ in order to trace back to where features common to *x* and *y* might have originated. Networks with $$mrca=\left( {\begin{array}{c}n\\ 2\end{array}}\right) $$ could be especially useful.
- $c=\vert Cl(N)\vert$ is the number of distinct clusters *cl*(*u*) for $u\in V$. In a tree (with no vertices of out-degree one) $c=v$. For a DCTC network $c=v-{\beta }(N)$ by Theorem 5.1.
- *vi* is the number of visible vertices. In a tree $vi=v$. By Corollary 4.6, $vi=v$ in a normal or DCTC network. It is useful for all vertices to be visible.
- 0*tc* is the number of non-leaf vertices with no tree-child. In a tree, $0tc=0$. In a tree-child network, by definition $0tc=0$. A network with $0tc>0$ is not tree-child. It will be useful for a network to have small 0*tc*.
- When several networks are being analyzed related to the network *N*, for the specified network *M*, $d=d_{RF}(N,M)$.

For example, in Fig. 5 we have $n=4$, $v=11$, $a=13$, $h=2$, $r=0$, $o1=1$, $o2=6$, $o2m=6$, $mrca=6$, $c=10$, $vi=11$, $0tc=0$.

## Examples

This section contains three detailed examples of the calculation of $\text {DCTC}(N)$ from a network *N*, two of them using real data.

We shall occasionally compare $\text {DCTC}(N)$ with $\text {Norm}(N)$ and $\text {FHS}(N)$. Recall that $\text {Norm}(N)$ was a uniquely determined normal network constructed from *N* as described in (Willson 2022).

The Francis et al. (2021) “normalization” of *N* (denoted here as $\text {FHS}(N)$) has vertex set the set of all visible vertices of *N*. There is an arc (*u*, *v*) in $\text {FHS}(N)$ between distinct vertices *u* and *v* provided there is a path in *N* from *u* to *v* and in addition there is no visible vertex *w* distinct from *u* and *v* such that there are paths from *u* to *w* and from *w* to *v*. It is proved in Francis et al. (2021) that $\text {FHS}(N)$ is a uniquely determined normal network. In general, there is no CSD map from *N* to $\text {FHS}(N)$.

**Fig. 12:**
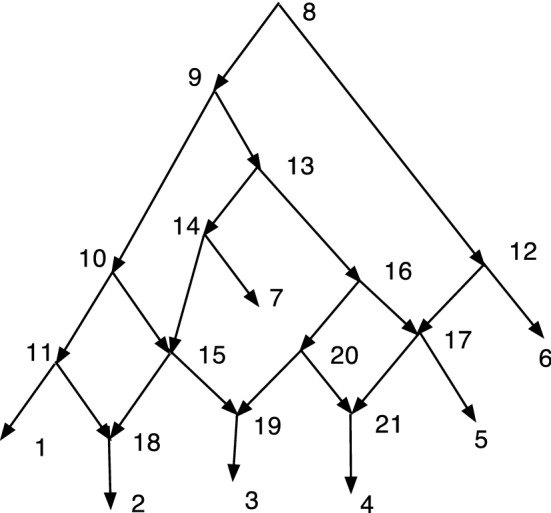
Vertex 15 is a tree-child obstacle of type 1 with two allowable 1-fold parent chains 10, 15 and 14, 15. Vertex 20 is a tree-child obstacle of type 2 with one allowable twofold parent chain 13, 16, 20

**Fig. 13:**
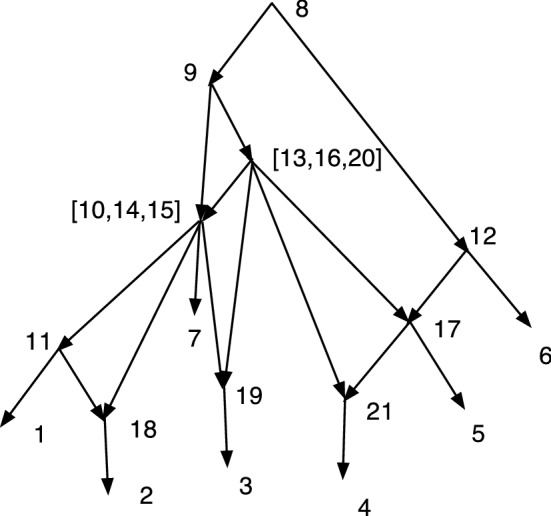
$M_D(N)$ for *N* in Fig. 12, where $D=\{(10,15), (14,15)$, $(13,16), (16,20)\}$. Note $cl(9)=cl([13,16,20])$, so $M_D(N)$ is not SCD

**Fig. 14:**
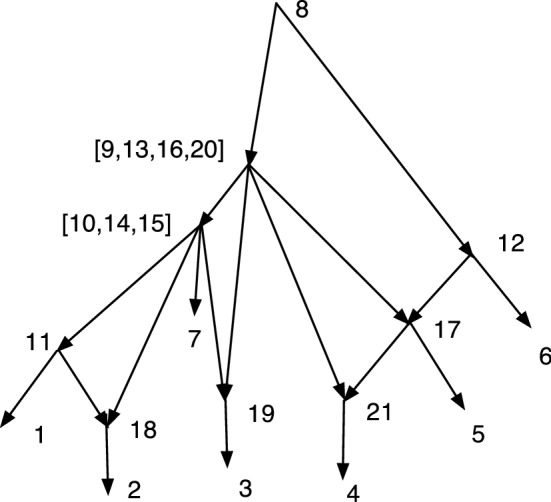
$N_2=\text {SCD}(M_D(N))$ for Fig. 12 after merging (9,[13,16,20]) into [9,13,16,20]. Since $N_2$ is tree-child, $\text {DCTC}(N)=N_2$ for *N* in Fig. 12

**Fig. 15:**
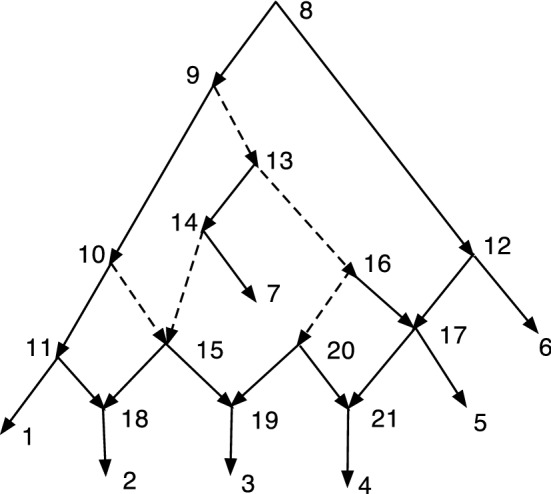
The wired lift of $\text {DCTC}(N)$ for *N* in Fig. 12. Dashed arcs correspond to identifications. Solid arcs represent arcs of $\text {DCTC}(N)$

### Example 1

Figure 12 shows a network *N* which is already SCD, so $N_1=N$. It has two tree-child obstacles 15 and 20. Vertex 15 has type 1 with two allowable onefold parent chains 10, 15 and 14, 15. Hence, $D15 = \{(10,15), (14, 15)\}$. Vertex 20 has type 2 with one allowable twofold parent chain 13, 16, 20; hence $D20=\{(13,16), (16,20)\}$. Note that 16, 20 is not an allowable 1-fold parent chain because 17 is hybrid. Then $D=D15\cup D20=\{(10,15), (14,15), (13,16), (16,20)\}$. *D* is strongly closed, and $M_D(N)$ is shown in Fig. 13. In this example, to construct $M_D(N)$ all that is needed is to contract each of the arcs in *D* into a point. From *D*, we know $10\sim 15\sim 14$ and those vertices are identified into a single vertex [10, 14, 15]. Similarly $13\sim 16\sim 20$.

Often when there is an allowable parent chain $p^k, p^{k-1}, \ldots , p^0=c$ for an obstacle *c*, then some of the $p^{k-1}, \ldots , p^1$ are also obstacles. This is not always true, however, as is seen from the parent chain 13, 16, 20, in which 16 is not an obstacle since it has the tree-child 20. This does not make 20 of type 1 since the tree-child of 16 is in the parent chain.

Let $\psi :N\rightarrow M_D(N)$ be the projection. Then $\psi (13)=\psi (16)=\psi (20)=[13,16,20]$. Similarly $\psi (10)=\psi (14)=\psi (15) = [10,14,15]$. For other vertices *v* of *N*, $\psi (v)=v$. In $M_D(N)$, $cl(9)=cl([13,16,20])$, so $M_D(N)$ is not SCD. The procedure then has us compute $\text {SCD}(M_D(N))$. To find $\text {SCD}(M_D(N))$, only (9,[13,16,20]) is merged into [9,13,16,20]. Figure 14 shows $N_2=\text {SCD}(M_D(N))$. Since $N_2$ is tree-child, we find $\text {DCTC}(N)=N_2$ and its height is 2. If now $\psi :N\rightarrow \text {DCTC}(N)$, we have, for example, $\psi ^{-1}([10,14,15])=\{10,14,15\}$. $N_2$ has three redundant arcs ([9,13,16,20],19), ([9,13,16,20],21), and ([10,14,15],18).

Figure 15 shows the wired lift $(\psi ^{-1}, E_1)$ of $\text {DCTC}(N)$ into *N*. In Fig. 15, $E_1$ consists of the solid arcs, and dashed arcs correspond to identifications. Thus, $10\sim 15\sim 14$ and $9\sim 13\sim 16\sim 20$ can be recognized from the dashed arcs, indicating that $\text {DCTC}(N)$ includes vertices [10,14,15] and [9,13,16,20]. Paths in $\text {DCTC}(N)$ correspond to g-paths in the wired lift. For example, there is no path in *N* from 10 to 7. There is, however, a path [10,14,15],7 in $\text {DCTC}(N)$, which corresponds to the g-path 10,15,14,7 in the wired lift since dashed arcs can be followed either forwards or backwards.

Table 1 compares a number of different networks related to Fig. 12.

**Table 1:** Comparison of networks related to *N* in Fig. 12. All have the same number $n=7$ of leaves. The number of 2-sets of leaves is 21. Other parameters are those discussed in Sect. 10. The networks $\text {DCTC}(N)$, $\text {Norm}(N)$, and $\text {FHS}(N)$ are all DCTC

Network	*v*	*a*	*h*	0*tc*	*r*	*c*	*o*1	*o*2	*o*2*m*	$\text {mrca}$	vi	*d*
*N*	21	25	5	2	0	18	3	11	11	21	18	0
$M_D(N)$	17	21	5	1	4	13	3	7	6	21	16	7
$\text {DCTC}(N)$	16	19	4	0	3	13	3	6	6	21	16	7
$\text {FHS}(N)$	17	19	3	0	0	15	2	8	8	20	17	3
$\text {Norm}(N)$	13	13	1	0	0	13	0	6	6	21	13	7

The normal network $\text {FHS}(N)$ has $o1=2$ vertices of out-degree one but it turns out that both such vertices have leaves as children. Hence $\text {SCD}(\text {FHS}(N))=S'(\text {FHS}(N)=\text {FHS}(N)$ is also DCTC by Corollary 8.2. Similarly $\text {Norm}(N)$ has $o1=0$ vertices of out-degree one so it is also DCTC.

Table 1 thus contains the three DCTC networks $\text {DCTC}(N)$, $\text {Norm}(N)$, and $\text {FHS}(N)$. There is a CSD map $\psi :N\rightarrow \text {DCTC}(N)$. There is, however, no CSD map from *N* to $\text {Norm}(N)$ (only a connected map, see Willson (2022)), and no CSD map from *N* to $\text {FHS}(N)$.

Of the three DCTC networks, $\text {DCTC}(N)$ as expected contains redundant arcs, and the others do not. Somewhat surprisingly it has 3 redundant arcs when *N* had none. It also contains the most hybrid vertices among the three DCTC networks, just one more than $\text {FHS}(N)$ but three more than $\text {Norm}(N)$.

From the $\text {mrca}$ column, in *N*, $\text {mrca}(x,y)$ exists for all $x,y\in X$ and this is also true for $\text {DCTC}(N)$ and $\text {Norm}(N)$. Surprisingly, Table 1 shows that for exactly one $\{x,y\}$, $\text {mrca}(x,y;\text {FHS}(N))$ does not exist; this turns out to be $\text {mrca}(2,3 )$.

We see that $\text {FHS}(N)$ is closer to the data set *N* in the sense of $d_{RF}$ than is $\text {DCTC}(N)$ because $d_{RF}(N,\text {FHS}(N)) <d_{RF}(N,\text {DCTC}(N))$. But $\text {DCTC}(N)$ contains an additional reticulation and three redundant arcs indicating where some specific modifications arose as *N* was simplified. Moreover, $\text {DCTC}(N)$ has a wired lift.

**Fig. 16:**
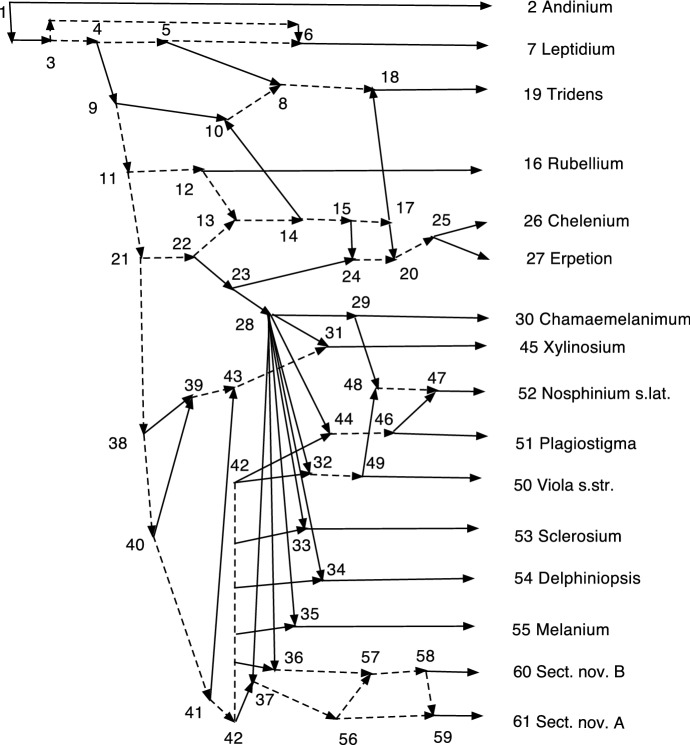
The wired lift for $\text {DCTC}(N)$ where *N* is the network for *Viola* in Marcussen et al. (2015). The line segment with both ends 42 is regarded as a single vertex. This wired lift differs substantially from the wired lift of $\text {Norm}(N)$ in Willson (2022)

### Example 2

Marcussen et al. in Marcussen et al. (2015) study the angiosperm genus *Viola* and present a phylogenetic network *N* with 16 leaves and with 21 proposed polyploid speciations in their Fig. 4. In our Fig. 16, we show the wired lift of $\text {DCTC}(N)$. If all the arcs are instead made solid, we obtain the network of Marcussen et al. (2015). Here we will sometimes abbreviate $\text {DCTC}(N)$ by $\text {DCT}$.

A normal network (which I denote $\text {FHS}(N)$) obtained as a simplification of *N* has been published in Francis et al. (2021) and another (called $\text {Norm}(N)$) in Willson (2022). Both differ substantially from $\text {DCT}$.

Table 2 makes a comparison among several networks related to *N*.

To find $\text {DCT}$, we first compute $\text {SCD}(N)$, for which the data are also shown in Table 2. It contains $0tc=3$ tree-child obstacles—one of type 1 with 1 allowable parent chain, one of type 1 with 2 allowable parent chains, and one of type 2 with one allowable parent chain, leading to *D* containing 5 arcs. Then $\text {SCD}(M_D(\text {SCD}(N)))$ is DCTC hence is $\text {DCT}=\text {DCTC}(N)$ with height 2.

**Table 2:** Comparison of networks related to *N* for $$ {Viola}$$ from Marcussen et al. (2015). All have the same number $$n=16$$ of leaves. The number of 2-sets of leaves is 120. None of the networks contain trivial vertices. Other parameters are those discussed in Sect. 10. The networks $$\text {DCTC}(N)$$, $$\text {Norm}(N)$$, and $$S'(\text {FHS}(N))$$ are DCTC networks

Network	*v*	*a*	*h*	0*tc*	*r*	*c*	*o*1	*o*2	*o*2*m*	$\text {mrca}$	vi	*d*
*N*	61	81	21	11	4	32	21	24	14	87	43	0
$\text {SCD}$	39	52	12	3	2	32	7	16	14	87	35	0
$\text {DCTC}$	32	42	10	0	9	26	6	10	10	120	32	6
$\text {Prenorm}$	35	47	11	1	10	27	7	12	11	120	34	5
$\text {Norm}$	29	31	2	0	0	27	2	11	11	120	29	5
$\text {FHS}$	31	34	3	0	0	28	3	12	12	118	31	4
$S'(\text {FHS})$	30	33	3	0	0	28	2	12	12	118	30	4

$\text {FHS}(N)$ is normal. By Corollary 8.2, $\text {SCD}(\text {FHS}(N))$ is DCTC, but it is more easily computed as $S'(\text {FHS}(N))$. From Table 1, $\text {FHS}(N)$ has $o1=3$ vertices with out-degree 1; these turn out to be 18, 20, 47 with respective children 19, 25, 52. Note that 19 and 52 are leaves but 25 is not, so only 25 needs to be merged with its parent to compute $S'(\text {FHS}(N))$. By Sect. 8, $S'(\text {FHS}(N))$ has one fewer vertex (of out-degree 1) from merging (20,25) and one fewer arc than $\text {FHS}(N)$. The other data are unchanged.

$\text {Norm}(N)$ has $o1=2$ vertices with out-degree 1. The child of both is a leaf. So $\text {SCD}(\text {Norm}(N)) = \text {Norm}(N)$ is SCD, hence DCTC by Corollary 8.2.

The table thus contains three DCTC networks: $\text {DCTC}(N)$, $\text {Norm}(N)$, and $S'(\text {FHS}(N))$.

It is interesting that $\text {SCD}(N)$ already shows a large reduction in the number of vertices, including a large reduction in the number of hybrid vertices, down to $h=12$. Most of the vertices with out-degree one have been eliminated; the remaining are hybrid vertices with out-degree one of form *p*(*x*) for $x\in X$, and ${\beta }(\text {SCD}(N))=o1=7$. As a result, $\text {SCD}(N)$ has only $0tc=3$ non-leaf vertices with no tree-child, instead of 11.

From the table, the 21 hybrids of *N* have been reduced to 10 reticulations in $\text {DCT}$. From Fig. 16, they are $8=10=18$, $20=25$, $32=49$, 33, 34, 35, $36=37$, $31=39=43$, $44=46$, $47=48$. All of them were hybrids in *N* but some of the hybrids of *N* have been merged. Hybrids 6, 13, 14, 57, 59 in *N* disappeared since they were merged with their parental line. For any DCTC *X*-network *M*, $o1={\beta }(M)$; hence ${\beta }(\text {DCT})=6$. From Fig. 16, the 6 post-hybrid leaves are 19, 45, 52, 53, 54, 55.

In $\text {DCT}$, the $o2m=10$ vertices of form $\text {mrca}(x,y;\text {DCT})$ are 1, [3,4,5,6], [9, 11, 12, 13, 14, 15, 17, 21, 22, 38, 40, 41, 42], 23, 24, 28, 29, [32, 49], [36, 37, 56, 57, 58, 59], [44, 46]. We recognize them in Fig. 16 since they are all the vertices of out-degree 2 or higher by Corollary 5.3. Since there are many identifications, it may be easier sometimes to just use one vertex label from each equivalence class. Some of these vertices are $\text {mrca}(x,y)$ for many different (*x*, *y*); for example, $28 =\text {mrca}(x,y;\text {DCT})$ for 41 different 2-sets $\{x,y\}$, such as $28=\text {mrca}(45,50)$. This is related to the fact that $outdeg(28;\text {DCT})=8$. In contrast, $44=\text {mrca}(51,52;\text {DCT})$ and there is no other 2-set $\{x,y\}$ satisfying $44=\text {mrca}(x,y;\text {DCT})$.

Moreover (which is not guaranteed in general as shown in Fig. 6) in $\text {DCT}$ for every pair (*x*, *y*) of distinct leaves, $\text {mrca}(x,y;\text {DCT})$ exists, as shown in Table 2 by $\text {mrca}= 120$.

A biologist might be interested in $\text {mrca}(x,y)$ in order to trace back to where features common to *x* and *y* might have originated. From Table 2, in *N*, 33 different $\{x,y\}$ have no $\text {mrca}(x,y;N)$. For example, $\text {mrca}(45,50;N)$ does not exist, while $28=\text {mrca}(45,50;\text {DCT})$. The use of $\text {mrca}(x,y;\text {DCT})$ when $\text {mrca}(x,y;N)$ does not exist can narrow the range of sources of common features of *x* and *y*.

In the wired lift, $E_1$ contains 49 solid arcs and 32 dashed arcs. Hence, some of the 42 arcs of $\text {DCT}$ are represented by more than one member of $E_1$. For example (14,10) and (17,18) represent the same arc of $\text {DCT}$ since 14 and 17 are identified (as indicated by dashed arcs), and similarly 10 and 18 are identified.

The networks $\text {Prenorm}(N)$ and $\text {Norm}(N)$ are described in more detail in Willson (2022), and $\text {FHS}(N)$ in Francis et al. (2021). $\text {Norm}(N)$ is constructed by removing the redundant arcs from $\text {Prenorm}(N)$.

In this example, $\text {Prenorm}(N)$ is not tree-child, since $0tc=1$.

Note that $\text {Norm}(N)$ has 3 fewer vertices, 11 fewer arcs, and 8 fewer reticulations than $\text {DCT}$. Thus, $\text {Norm}(N)$ contains substantially less information about the dataset than does $\text {DCT}$. The loss of the hybrids and arcs is largely because many were associated with the 10 redundant arcs that were removed from $\text {Prenorm}(N)$ to make $\text {Norm}(N)$.

It is interesting that $\text {FHS}(N)$ has only 3 hybrid vertices, since its construction does not involve $\text {SCD}$, which made an initial large drop in hybrid vertices for the calculation of both $\text {DCT}$ and $\text {Norm}(N)$.

From the table, we see that $\text {mrca}(x,y;S'(\text {FHS}(N)))$ exists for all $x,y\in X$ except for two choices. These choices are $\{x,y\}=\{19,26\}$ and $=\{19,27\}$. For example, $12\le 19$ and $12\le 26$ and 12 almost satisfies the condition for being $\text {mrca}(19,26;S'(\text {FHS}(N)))$; yet $22\le 19$ and $22\le 26$ but it is false that $22\le 12$.

Also from the table, $d_{RF}(N,\text {DCT})=6$, $d_{RF}(N,\text {Norm}(N))=5$, and $d_{RF}(N,$ $S'(\text {FHS}(N)))=4$. Hence, $\text {DCT}$ is not as close an approximation to *N* as either of the others in terms of $d_{RF}$. $\text {DCT}$ has the advantage over $\text {Norm}(N)$ and $S'(\text {FHS}(N))$ of many more confirmed hybrid vertices and many more arcs. Moreover, there is a CSD map $\psi :N\rightarrow \text {DCT}$. In contrast, $\text {Norm}(N)$ has only a connected map $\psi :N\rightarrow \text {Norm}(N)$, which is not as strong a condition. $\text {DCT}$ also has the advantage over $\text {Norm}(N)$ of a significantly larger set $E_1$ (rather than the many dashed arcs in $\text {Norm}(N)$ to avoid redundant arcs).

In Sect. 12, we will make some further comments about Example 2.

### Example 3

**Fig. 17:**
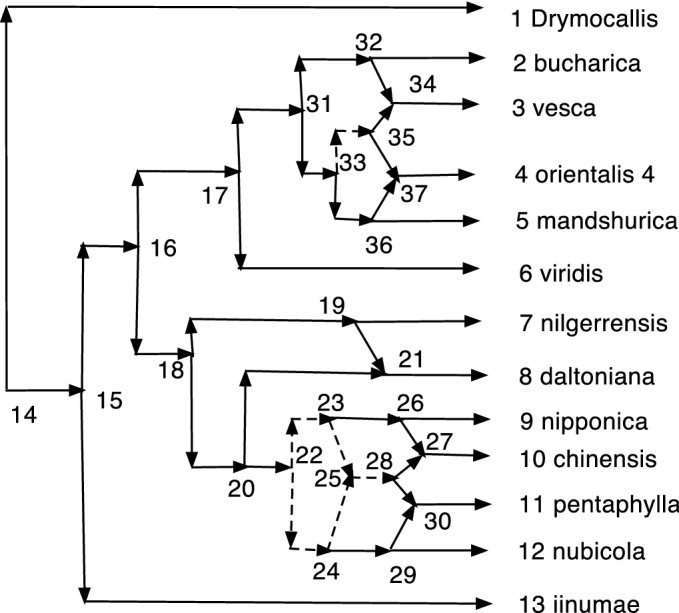
The wired lift for $\text {DCTC}(N)$ where *N* is the network in Kamneva et al. (2017), Figure S7–9a, concerning strawberries (*Fragaria*). If all the arcs are solid, we obtain their figure S7-9a. The dashed arcs indicate identifications of vertices, so [22,23,24,25,28] is one vertex of $\text {DCTC}(N)$. The solid arcs represent arcs of $\text {DCTC}(N)$. For example, [22,23,24,25,28] has tree-children 26 and 29

Here is another example with real data. Kamneva et al. (2017) study allopolyploid origins in strawberries (*Fragaria*). In their Additional File 4, Figure S7–9a is the cluster network for their dataset 9, constructed using all fragments passing the SH test against 100 random trees by support at least 15%. Let *N* be the network of their Figure S7–9a. The wired lift of $\text {DCTC}(N)$ is shown in our Fig. 17. Table 3 compares some networks related to *N*.

There are 13 leaves with Drymocallis being the outgroup that roots the network. $\text {SCD}(N)$ is found by merging the single arc (25,28) into the vertex [25,28]. There are two obstructions [25,28] and 35 each of type 1, where [25,28] has 2 allowable parent chains and 35 has one. Then, $D=\{(23,[25,28]), (24,[25,28])$, $(33, 35)\}$. After finding $M_D(\text {SCD}(N))$, we must merge the arc (22, [23, 24, 25, 28]) to obtain $\text {DCTC}(N)$. Its wired lift is shown in our Fig. 17. Table 3 compares information about some relevant networks.

**Table 3:** Comparison of networks related to *N* for Figure S7–9a from Kamneva et al. (2017) concerning strawberries. Abbreviations for the columns are the same as in Sect. 10. All networks have the same 13 leaves. The number of 2-subsets of leaves is 78. The networks $\text {DCTC}(N)$, $\text {Norm}(N)$, and $\text {FHS}(N)$ are all DCTC

Network	*v*	*a*	*h*	0*tc*	*r*	*c*	*o*1	*o*2	*o*2*m*	$\text {mrca}$	*vi*	*d*
*N*	37	42	6	2	0	31	6	18	18	78	34	0
$\text {SCD}(N)$	36	41	6	2	0	31	5	18	18	78	34	0
$\text {DCTC}(N)$	32	36	5	0	3	27	5	14	14	78	32	4
$\text {Norm}(N)$	29	30	2	0	0	27	2	14	14	78	29	4
$\text {FHS}(N)$	33	36	4	0	0	31	5	19	19	77	33	2

It is clear that $S'(\text {FHS}(N))=\text {FHS}(N)$ and $S'(\text {Norm}(N))=\text {Norm}(N)$, so by Corollary 8.2 both $\text {FHS}(N)$ and $\text {Norm}(N)$ are SCD hence are DCTC *X*-networks. There is, however, no CSD map from *N* to $\text {FHS}(N)$ or to $\text {Norm}(N)$.

From Table 3, we see that $\text {DCTC}(N)$ retains 5 out of the 6 hybrids of *N*, while $\text {FHS}(N)$ is next best with 4 hybrids. The hybrids of $\text {DCTC}(N)$ are 21, 27, 30, 34, 37 which were also hybrids of *N*. The other hybrid 25 of *N* was merged with its parental line and descendant 28 into [22, 23, 24, 25, 28].

Note that $\text {FHS}(N)$ is closest to *N* in terms of the distance $d_{RF}$ but lacks a CSD map and a wired lift. $\text {Norm}(N)$ as a DCTC network is inferior to $\text {DCTC}(N)$ since it has fewer vertices and fewer hybrids. The $r=3$ redundant arcs in $\text {DCTC}(N)$ are ([22,23,24,25,28],27), ([22,23,24,25,28],30), and ([33,35],37), all of which are parents of a hybrid. For example, the redundant arc ([22, 23, 24, 25, 28], 27) suggests that 27 arises from a hybridization of 26 with a species in the region [22, 23, 24, 25, 28].

## Discussion

Given a general network *N*, we have seen that $\text {DCTC}(N)$ has interesting mathematical properties. The biological significance of such a network $\text {DCTC}(N)$, however, is less clear.

The construction of $\text {DCTC}(N)$ usually involves two operations: The first is the calculation of $\text {SCD}(N)$, and the second is the calculation of $M_D(\text {SCD}(N))$ where *D* consists of the arcs of some allowable parent chains. We here consider these two operations separately.

**Fig. 18:**
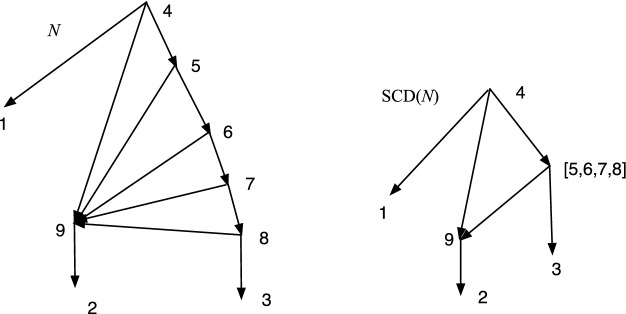
A network *N* on the left and $\text {SCD}(N)$ on the right. In *N*, 9 has in-degree 5

**Fig. 19:**
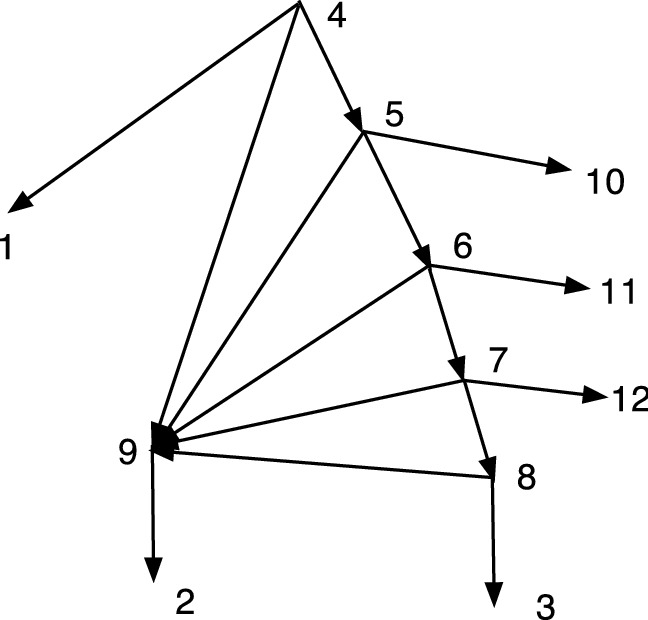
Adding more leaves to the network *N* of Fig. 18 retains the ladder and indicates the order of the vertices using information on the leaves. This network *M* is DCTC

**Fig. 20:**
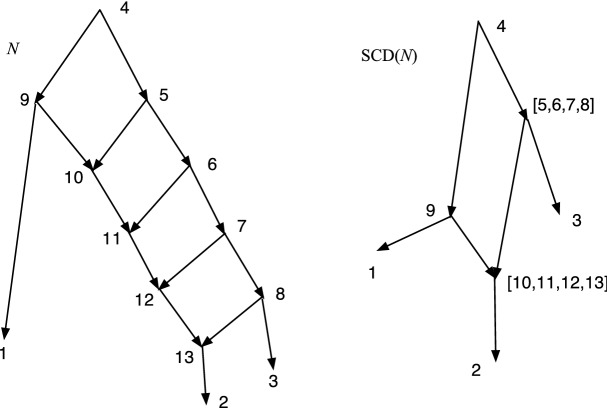
A network *N* on the left and $\text {SCD}(N)$ on the right. There is a “ladder” of vertices with hybrid children

To study the first operation, consider Fig. 18 showing a network *N* on the left with $\text {SCD}(N)$ on the right. In *N*, there is a “ladder” structure involving 4, 5, 6, 7, and 8. Note that $cl(5)=cl(6)=cl(7)=cl(8)=\{2,3\}$. Hence, in $\text {SCD}(N)$, the arcs (5, 6), (6, 7) and (7, 8) are contracted, so that 5, 6, 7, and 8 are all identified into the single vertex [5, 6, 7, 8]. This simplification recognizes the difficulty of distinguishing these vertices using only the data on the leaves, since those data usually consist largely of the genomes at the leaves. All that distinguishes them is their placement compared to the root 4. The contribution of 5 cannot readily be distinguished from the contribution of 6 since they both impact exactly the same leaves. While *N* may be correct in the sense that there might have been several stages of contribution to the genome of 9, nothing really identifies the relevant species. The result would be indistinguishable from the result if the species 5, 6, 7, and 8 were permuted. It can be argued that the data do not really support a network with distinct vertices 5, 6, 7, and 8; the simplification of *N* into $\text {SCD}(N)$ is an appropriate indication of what is really justified.

The fact that these identifications occur in $\text {DCTC}(N)$ also suggests a remedy: the addition of more leaves can separate 5, 6, and 7, as shown in the network *M* of Fig. 19. In *M*, $cl(5)=\{2,3,10,11,12\}$ and $cl(6)=\{2,3,11,12\}$ so $cl(5)\ne cl(6)$. In fact, *M* is DCTC, so $\text {DCTC}(M)=M$. Similar results could occur even if the arcs to the new leaves were instead replaced by tree-child paths to new leaves. Of course, finding the new leaves could be difficult; indeed, it is possible that there are no extant descendants of 5, 6, or 7 by tree-child paths if there were many extinction events along such lines of descent.

Similar considerations apply to a more complicated situation as in Fig. 20. In the network *N* on the left, there is another “ladder” $cl(5)=cl(6)=cl(7)=cl(8)=\{2,3\}$, and $cl(10)=cl(11)=cl(12)=cl(13)=\{2\}$. It is quite possible that permutations of 10, 11, 12, 13 and of 5, 6, 7, 8 could yield the same data on the leaves. Hence, $\text {SCD}(N)$, shown on the right, indicates this ambiguity in the interpretation of *N*. As for Fig. 18, this ambiguity could be resolved by adding new suitably placed leaves in *N*.

**Fig. 21:**
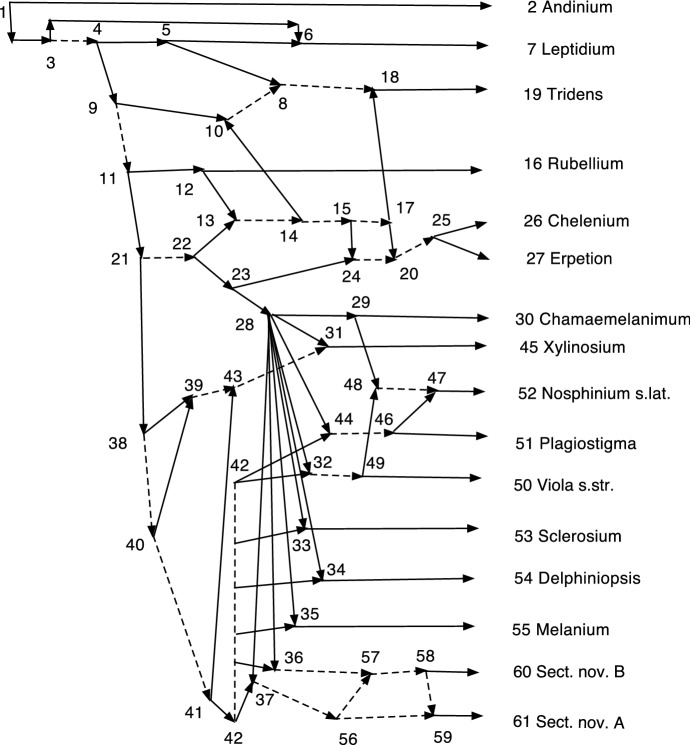
A wired lift of $\text {SCD}(N)$ where *N* is the network for the *Viola* data set (Marcussen et al. 2015) of Example 2

Less extreme “ladders” can occur. Figure 21 shows a wired lift of $\text {SCD}(N)$ where *N* is the *Viola* dataset for Example 2 of Sect. 11. There is a ladder involving 36, 37, 56, 57, 58 all with the same cluster $\{60,61\}$. There is a smaller ladder involving 38, 40, and 41; and another involving 15, 17, 20, 24. The merging of arcs indicates, for example, that more leaves would be needed to clarify the difference between vertices such as 56 and 57.

Figure 21 also shows several hybrid vertices *a* in *N* with a unique child *b* which is not a leaf, so $$cl(a)=cl(b)$$ and *a* and *b* are not distinguishable from the data; these include $$a= $$10, 13, 20, 24, 32, 37, 39, 43, and 48. In *N*, such arcs (*a*, *b*) may merely indicate the distinction between a hybrid vertex *a* and the next descendant *b* where another speciation event occurs. If *b* is a tree-child, as in the case $$a=13, b=14$$, then the merging is merely an artifact of our definition of SCD (so that only leaves $$\phi (x)$$ can be the unique child of a hybrid vertex *p*(*x*)) and these can be mentally retained. When *b* is hybrid, as for example in the case $$a=48, b=47$$, then *a* and *b* are parts of another ambiguous ladder, justifying the identification of 47 and 48.

We see that in general the merging of arcs from *N* in the formation of $\text {SCD}(N)$ identifies some ambiguities in interpreting the original network. When the networks are SCD, ambiguities of that sort are not present.

**Fig. 22:**
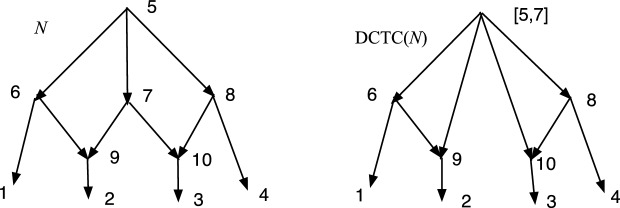
An SCD network *N* on the left and $\text {DCTC}(N)$ on the right. Note that 7 is an obstacle of type 1 and is not visible

**Fig. 23:**
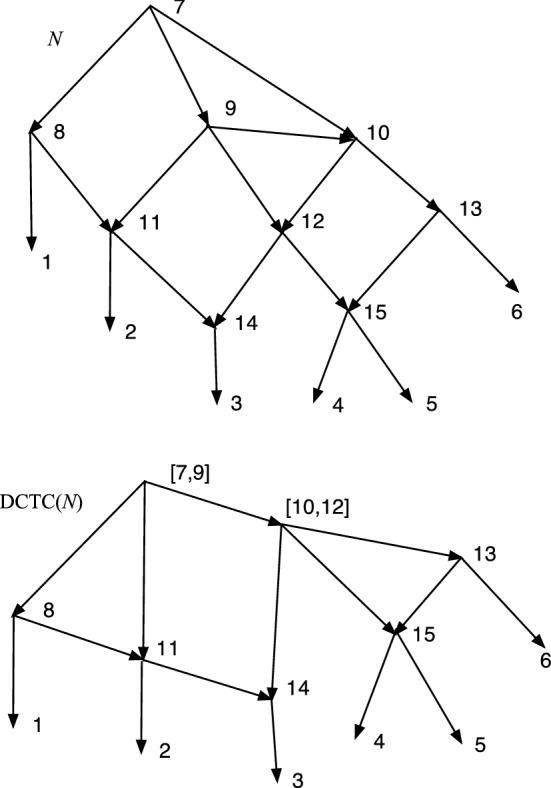
An SCD network *N* on the top and $\text {DCTC}(N)$ below

The interpretation of the second operation (finding $M_D(\text {SCD}(N))$ where *D* consists of the arcs of some allowable parent chains to make the network tree-child) is different. Figure 22 shows an SCD network *N* on the left and also $\text {DCTC}(N)$ on the right. In this case 7 is an obstacle of type 1 with allowable parent chain 5, 7. In $\text {DCTC}(N)$ the arc (5, 7) has therefore been contracted to a point [5, 7]. Note that in *N*, $cl(5) = \{1,2,3,4\}$ while $cl(7)=\{2.3\}$. As a result, the networks *N* and $\text {DCTC}(N)$ differ in their resultant statistical effects on the genomes. For example, if *N* is assumed to describe the genetic history, a mutation from 5 which is absent in 1 and 4 but present in 2 and 3 should be more common than if instead $\text {DCTC}(N)$ is assumed.

In general, if $p^k, p^{k-1}, \dots , p^1, p^0=c$ is an allowable parent chain for an obstacle *c* of type *k*, it is immediate that for each $i\le k$, $cl(p^i;N) \subseteq cl([p^k,\ldots ,c])$, where the latter is interpreted in $\text {DCTC}(N)$. Any inheritance suggested by *N* is also possible in $\text {DCTC}(N)$, while $\text {DCTC}(N)$ has additional possibilities and the statistics of the mutations may have changed. The question is whether the additional properties of $\text {DCTC}(N)$ are sufficiently useful to justify the change.

Recall that a vertex *v* in *N* is *visible* to a leaf $\phi (x)$ for $x\in X$ if every path from the root $\rho$ to $\phi (x)$ contains *v*. Thus, the genome at *v* is very likely to affect the genome of each such $\phi (x)$. Note that in *N* of Fig. 22, 7 is not visible. This fact makes the influence of 7 in *N* on the genomes of the leaves hard to interpret. By way of contrast, every vertex of $\text {DCTC}(N)$ is visible. In $\text {DCTC}(N)$, 6 is visible to 1, 9 to 2, 10 to 3, and 8 to 4, while [5,7] is visible to all leaves and each leaf is visible to itself. Each vertex has at least one leaf to which it is visible.

Figure 23 shows another SCD network *N* and below it $\text {DCTC}(N)$. Note that 9 and 12 are not visible. Moreover, they are obstacles of type 1 with allowable parent chains 7, 9 and 10, 12, respectively. The effects of 9 and 12 on the genomes at the leaves are difficult to understand because there are several possibilities for the inheritance of the genomes. In contrast, every vertex of $\text {DCTC}(N)$ is visible. The root [7,9] is visible to every leaf, and [10,12] is visible to 4, 5, and 6; 13 is visible to 6; 15 to 4 and 5; 8 to 1; 11 to 2; 14 to 3.

Genetic influence is easiest to interpret making use of tree-child paths. Note that there is no tree-child path in *N* from 7 or 9 to 6, but the path [7,9], [10,12], 13, 6 is a tree-child path in $\text {DCTC}(N)$.

Consider again the *Viola* dataset *N* analyzed in Example 2 and Figs. 16 and 21. From Table 2, only 43 of the 61 vertices of *N* are visible, and only 35 of the 39 vertices in $\text {SCD}(N)$ are visible. The four vertices of $\text {SCD}(N)$ (Fig. 21) which are not visible are 5, [13,14,15,17], 42, and [38,40,41], which are therefore problematic. In $\text {DCTC}(N)$ (Fig. 16) 5 has been merged into [3,4,5,6], while each of the others has been merged into [9,11,12,13,14,15,17,21,22,38,40,41,42], making them visible.

For the *Fragaria* dataset *N* of Kamneva et al. (2017) and Example 3, Fig. 17, $\text {SCD}(N)$ merely identifies 25 and 28 since $cl(25)=cl(28)$. But then [25,28] is not visible, so $\text {DCTC}(N)$ contains the vertex [22, 23, 24, 25, 28]. In Fig. 17, this looks merely like a complicated ladder being identified.

In general, $\text {DCTC}(N)$ is a network in which minimal simplifications have been made to *N* so that every vertex becomes visible. As a result, every vertex has at least one leaf on which its genetic influence is important. Changes from $\text {SCD}(N)$ to $\text {DCTC}(N)$ indicate failures of vertices in $\text {SCD}(N)$ to be visible.

## Data Availability

Not applicable.
